# Pair distribution function “feat.” solid-state NMR in a combined approach for structure determination of organic molecules

**DOI:** 10.1038/s42004-026-02233-x

**Published:** 2026-10-06

**Authors:** Chiara Rosso, Toms Rekis, Michele R. Chierotti, Federica Bravetti

**Affiliations:** 1 Department of Chemistry and NIS Center, University of Turin, Turin, Italy; 2 Institute of Inorganic and Analytical Chemistry, Goethe University, Frankfurt am Main, Germany

**Keywords:** Method development, X-ray diffraction, NMR spectroscopy

## Abstract

Investigating the local structure of molecular solids is critical for establishing structure–property relationships and controlling material performance. Pair distribution function (PDF) analysis probes poorly crystalline, nanocrystalline and amorphous materials, yet its application to organic compounds remains limited. The recently developed Global-PDF-Fit method enables ab initio crystal structure determination of organic molecules, although intrinsic limitations can complicate unique structure identification. Here, we present a synergistic workflow integrating Global-PDF-Fit with solid-state NMR (SSNMR), demonstrating how restraints and intermolecular proximity constraints from 1D magic angle spinning (MAS) and 2D ^1^H-^13^C double cross polarization and ^1^H double quantum/^1^H single quantum MAS SSNMR experiments reduce computational time, guide structure ranking, and increase reliability, enabling unambiguous structure determination. Demonstrated on 5-aminosalicylic acid and S-naproxen (crystallite sizes of 50–55 nm), the approach provides a robust strategy for structure determination and represents a significant advance in the field of local-structure elucidation of poorly crystalline and nanocrystalline organic solids, where conventional diffraction techniques fail.

**Figure:**
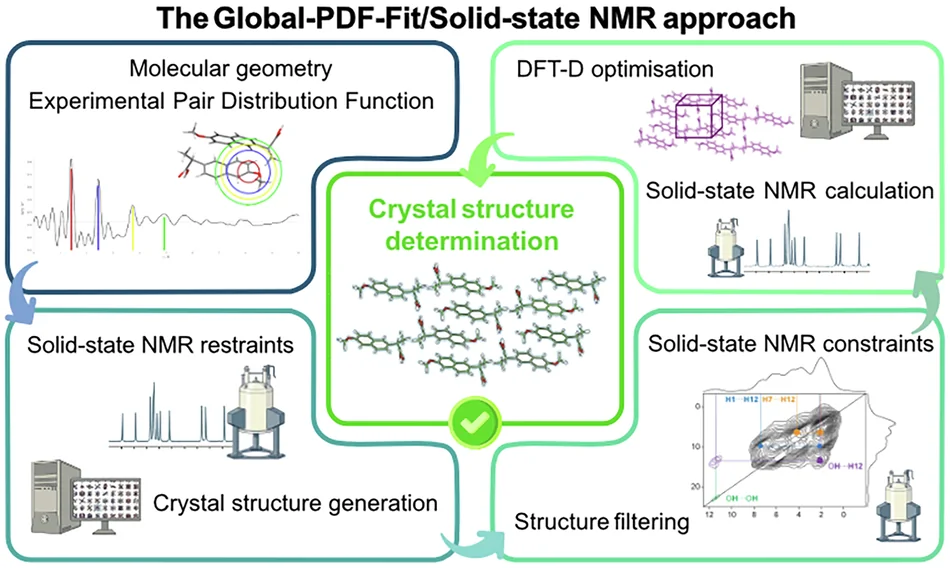

## Abstract (web summary)

Pair distribution function (PDF) analysis provides a valuable approach for probing poorly crystalline, nanocrystalline or amorphous materials, but its application to organic compounds remains underexplored. Here, the authors present a workflow that integrates the Global-PDF-Fit method with solid-state NMR to reduce computational time and guide structure ranking, facilitating unambiguous structure determination as demonstrated for 5-aminosalicylic acid and S-naproxen

## Introduction

Understanding the solid-state structures of molecular compounds is essential for establishing structure–property relationships and for the rational tuning of material performance, as exemplified by crystal engineering^1^. This need is particularly pronounced in the pharmaceutical field, where key properties of an Active Pharmaceutical Ingredient (API), including solubility, dissolution rate, storage stability, and tabletability, are strongly influenced by its solid-state form and structure^2–4^. Moreover, structure and solid form variations arising from synthesis and storage conditions can affect drug stability and bioavailability, posing challenges for regulatory compliance and quality control^5–7^.

A broad range of analytical techniques is available for crystal structure elucidation, spanning well-established and emerging methods. Single-crystal X-ray diffraction (SCXRD) remains the gold standard; however, it requires single crystals of sufficient size (typically 50–250 μm). In the pharmaceutical field, growing such crystals is often challenging, as many solid forms of drug candidates are obtained *via* mechanochemical techniques. While these approaches enable greener, solvent-minimizing syntheses, they generally yield micro- or nanocrystalline powders that are unsuitable for SCXRD^8^. Electron diffraction can, in principle, address small crystallite sizes (typically down to ~50 nm)^9^, but it is often unsuitable for organic compounds with very small crystallite sizes due to beam sensitivity, limited vacuum stability, and thermal decomposition or sublimation during measurement^10^. Structure determination from powder X-ray diffraction (PXRD) provides an alternative, as data can be collected under variable temperature or pressure conditions across all classes of compounds. However, its reliability and applicability decreases when diffraction patterns are affected by diffuse scattering and peak broadening and overlap due to disorder or reduced crystallite size^11^. Indeed, indexing a powder diffraction pattern usually requires at least 20 well separated peaks^12^. In the case of poorly crystalline samples, the number of resolved peaks decreases drastically^13^, preventing indexing and thus structure solution with direct- or indirect-space methods. This limitation was circumvented by Habermehl et al.^14^, with the development of the FIDEL-GO (FIt with DEviating Lattice parameters - Global Optimization) program for structure solution and refinement, starting from unindexed powder data. However, in the case of nanocrystalline samples with a crystallite size lower than 40 nm, with only a few broad peaks in the 0–30 2θ region, the method reaches its limitation, as the powder patterns do not contain enough information to properly fit the structural models.

In recent decades, pair distribution function (PDF) analysis, derived from total scattering data, has emerged as a valuable tool for probing the local structure of poor crystalline, nanocrystalline or amorphous materials by investigating interatomic distances^15^. Although PDF analysis is well established for inorganic compounds, such as liquids, glasses, nanocrystalline, amorphous or disordered inorganic materials^16–21^, its application to organic compounds has long remained limited due to intrinsic limitations. A major advanced in this field has recently been introduced by Schlesinger et al. with the development of the Global-PDF-Fit method^22^, based on the original FIDEL-GO program^14^. This approach enables the ab initio solution and refinement of organic crystal structures by a global fit to the experimental PDF without prior knowledge of lattice parameters or space group symmetry. The PDF of a nanocrystalline sample contains many more peaks that can be used to compare, fit and evaluate structural models, even when the powder diffractogram contains only two or three broad peaks. Experimental PDFs can be obtained from carefully measured powder patterns, with either short-wavelength synchrotron radiation (λ ~ 0.16–0.20 Å for high resolution) or more common laboratory sources (e.g., Cu, Mo, Ag). The Global-PDF-Fit method requires, beside a molecular geometry, an experimental PDF from carefully collected synchrotron powder pattern. Synchrotron radiation is required because it allows *Q*_*max*_ values above 20 Å^−^^1^ to be reached, providing the high resolution that cannot be achieved using conventional laboratory sources, such as Cu or Mo radiation^15^. This method was developed and successfully validated on crystalline, rigid organic molecules^22,23^. However, depending on the quality of the experimental data, the Global-PDF-Fit approach may yield several structural models that fit the experimental PDF data equally well, complicating the identification of a unique solution. This limitation has been reported for nanocrystalline samples of monomethyl-quinacridone (C_21_H_14_N_2_O_2_)^24^ and 4,11-difluoroquinacridone (C_20_H_10_F_2_N_2_O_2_)^25^. It was also observed that decreasing crystallite size reduces the reliability of the ${R}_{{wp}}^{{PDF}}$ value used to rank candidate structures after PDF fitting^26^. Consequently, the correct crystal structure may not appear among the top-ranked models and can be difficult to identify. In such cases, reliable structure selection requires combination with complementary techniques, most notably solid-state nuclear magnetic resonance (SSNMR).

The combination of SSNMR spectroscopy with diffraction-based methods and computational approaches (e.g., Crystal Structure Prediction (CSP)) is already well-established^27–31^, due to the capability of this technique of providing site-specific insights into the local structure of solid organic compounds, even in nanocrystalline (with small crystallite sizes, below 40 nm) and amorphous systems^32^. One-dimensional (1D) spectra (e.g., ^1^H magic angle spinning (MAS), ^13^C and ^15^N cross polarization magic angle spinning (CPMAS)) can offer insights on the number of symmetrically independent molecules in the unit cell (*Z*’), on the protonation and tautomeric states^23,33–37^, and the hydrogen-bond strength through characteristic chemical shifts^38^. Bi-dimensional (2D) experiments (e.g., ^1^H-^13^C heteronuclear correlation (HETCOR), ^1^H-^14^N heteronuclear multiple quantum coherence (HMQC), ^1^H double quantum (DQ)/^1^H single quantum (SQ) MAS) can, instead, provide information on intra- and intermolecular atom–atom proximities up to 3.5–4 Å for ^1^H–^1^H and up 3–4 Å for ^1^H–X^38–40^, enabling assessment of hydrogen-bond networks and molecular conformation in the solid-state. In some cases, even the measurement of precise intermolecular atom–atom distances are possible (e.g., in hydrogen bonds)^41–43^. Furthermore, comparison between computed and experimental SSNMR chemical shifts has become a well-established approach for validating structural models^29,44–46^, owing to the high sensitivity of SSNMR parameters to the local atomic environment.

Here, we present a synergistic workflow that integrates the Global-PDF-Fit method with SSNMR, highlighting how SSNMR information can be used as restraints and constraints to dramatically reduce computational time, improve structure ranking, and increase the reliability of the resulting crystal structures, ultimately enabling the identification of a unique and correct crystal structure. The method is demonstrated on two organic compounds, 5-aminosalicylic acid and S-naproxen, with a calculated crystallite size of ∼50 and ∼55 nm, respectively. More broadly, it offers an alternative strategy for determining the crystal structure of molecular solids, particularly in cases where conventional approaches are challenging or unfeasible, e.g., for nanocrystalline organic compounds with a crystallite size below 40 nm. Furthermore, the presented structure-filtering strategy can be applied independently of the structure-generation method to significantly reduce the number of inherently wrong crystal structures within a timeframe of a few minutes.

## Result and Discussion

### Combination of the Global-PDF-Fit approach with SSNMR

**Scheme 1:**
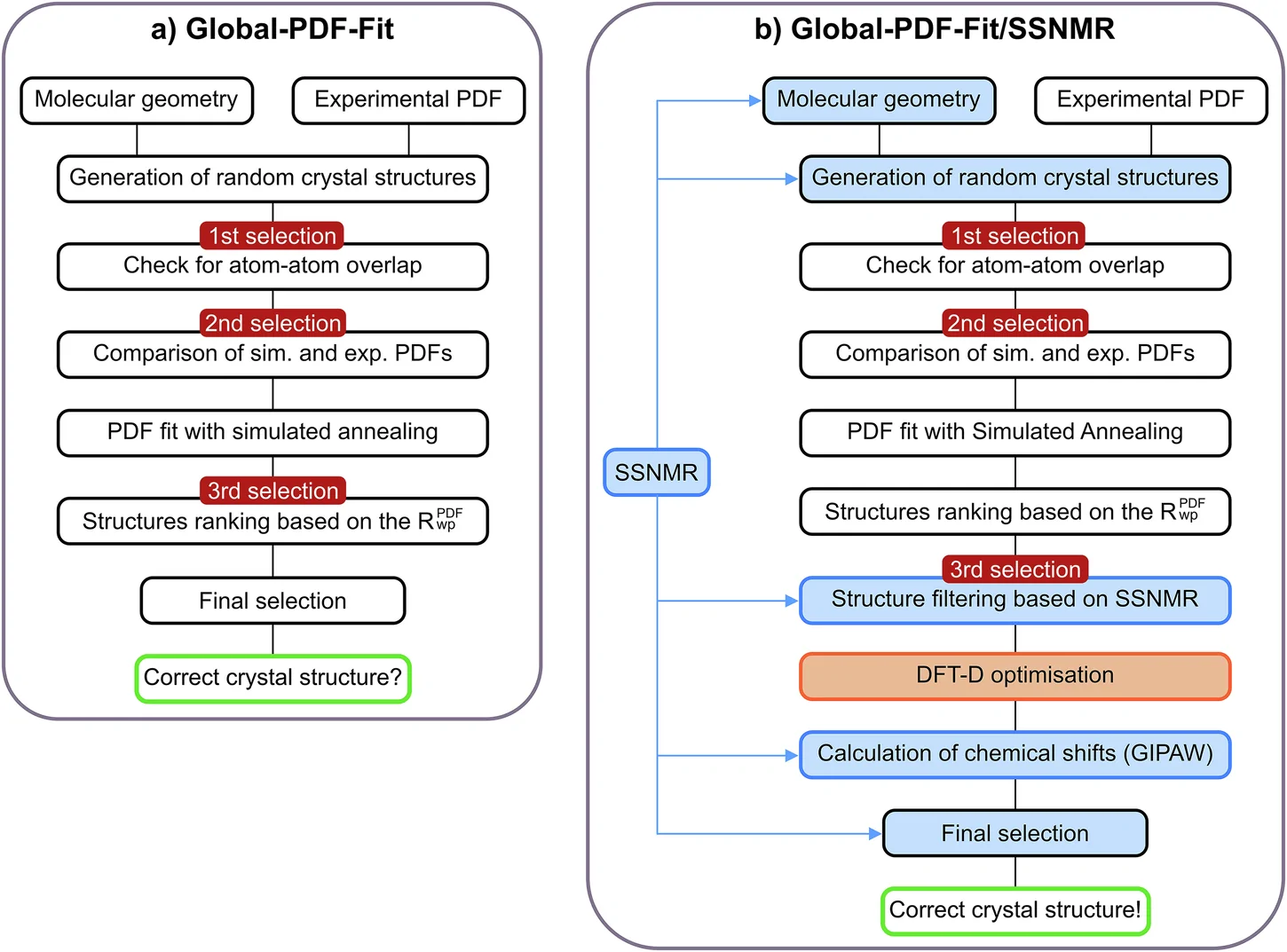
Flowchart of the Global-PDF-Fit method. **a** Original Global-PDF-Fit method; (**b**) combined Global-PDF-Fit/SSNMR approach. The contribution of SSNMR information is highlighted in light blue.

The Global-PDF-Fit approach is implemented in the program FIDEL-GO, originally developed by Habermehl et al. for structure determination from powder data, and subsequently adapted to PDF data^14,47^. In the present work, the original workflow (Scheme 1a) was adapted to integrate all available SSNMR information, thereby improving the molecular input, the structure generation, and the final selection process. The integration of the SSNMR data addresses inherent limitations of the original procedure, including its computational cost and the previously encountered challenge of identifying a unique structural solution. An overview of the combined Global-PDF-Fit/SSNMR workflow is given in Scheme 1b.

As for the original procedure, the Global-PDF-Fit/SSNMR approach begins with the generation of a large set of random starting structures (∼10^6^–10^8^) in various space groups, using a molecular geometry (created with the Mogul software^48,49^) as required input. At this initial stage, the SSNMR-derived information can be directly used as structural restraints to define the molecular geometry, e.g., to assess the correct protonation or tautomeric state, to identify intramolecular hydrogen bonds, or to obtain information about molecular conformations^50^. Furthermore, structure generation requires specifying two additional parameters: space groups and the number of independent molecules within the unit cell (*Z*’). Space groups are selected according to their frequency within the corresponding compound class, considering the molecular symmetry^51^. Concerning *Z*’, instead, several values should be tested. However, SSNMR enables an unambiguous determination of *Z*’, since symmetrically equivalent molecules are also magnetically equivalent and give rise to a single set of NMR signals for each molecule in the asymmetric unit^29^. This significantly reduces the computational effort required for the search^36,52^.

After the structure generation, the candidate structural models are firstly checked for atomic overlap (1^st^ selection, Scheme 1) and then compared to the experimental PDF using cross-correlation functions (2^nd^ selection, Scheme 1)^14,53^. At this point, the most promising structures are fitted to the experimental PDF in an elaborated multi-step process, which includes Simulated Annealing, using TOPAS^54^, and ranked according to their ${R}_{{wp}}^{{PDF}}$ values, which quantify the agreement of the structural models with the experimental PDF. The key innovation of the Global-PDF-Fit/SSNMR approach lies in the structure filtering following the PDF fit. Indeed, here, intermolecular atom–atom spatial proximities derived from 2D SSNMR experiments are used to filter out structurally inconsistent models. Whenever an intermolecular atom–atom proximity is detected by SSNMR, a corresponding proximity range between those atoms in the crystal structure is defined and applied as constraint; multiple observed proximities yield the definition of multiple ranges. During this step, careful interpretation of the SSNMR data is essential to avoid overinterpretation. In particular, only correlations unambiguously arising from intermolecular proximities should be considered, while those that might be due also to intramolecular ones must be excluded. For this reason, a detailed analysis of intramolecular distances is fundamental. For rigid or semi-rigid molecules, this analysis is straightforward, whereas flexible molecules require additional conformational analysis. In long-range ^1^H-^13^C Double-CP experiments, for instance, correlations may arise from both covalently bonded and non-bonded ^1^H–^13^C atom pairs. Consequently, a strong peak could be due either to a short intramolecular correlation, an intermolecular proximity, or both. In certain cases, these ambiguities can be ruled out based on the molecular structure and conformation of the compound. On the other hand, ^1^H–^1^H proximities in a 2D ^1^H DQ/^1^H SQ MAS spectra allow for a more definitive identification of intermolecular proximities between two symmetrically equivalent protons, e.g., H1 of molecule A and H1 of molecule B^39^. It is worth noting that the absence of correlations can itself provide valuable structural information. Therefore, careful evaluation is required, and information from different SSNMR spectra should be combined to build a reliable and comprehensive overview of the atomic-level environment. This SSNMR-guided selection step allows to drastically reduce the number of plausible structural models, enabling a more efficient and, most of all, reliable identification of the correct crystal structure. In other words, the original approach may produce structural models that exhibit low ${R}_{{wp}}^{{PDF}}$ values but remain inconsistent with experimental observations. By contrast, the SSNMR-filtering step allows such structural solutions to be excluded. The final selection of the correct crystal structure is achieved through lattice-energy optimisations by dispersion-corrected density functional theory (DFT-D) and gauge-including projector-augmented wave (GIPAW) SSNMR chemical shifts calculations. The correct structure is defined as that which exhibits: (i) good agreement between computed and experimental SSNMR chemical shifts, i.e., consistent ^1^H and ^13^C root-mean square errors (RMSEs); (ii) no or minimal geometric deviations upon DFT-D optimisation; and (iii) low relative lattice energy.

**Fig. 1:**
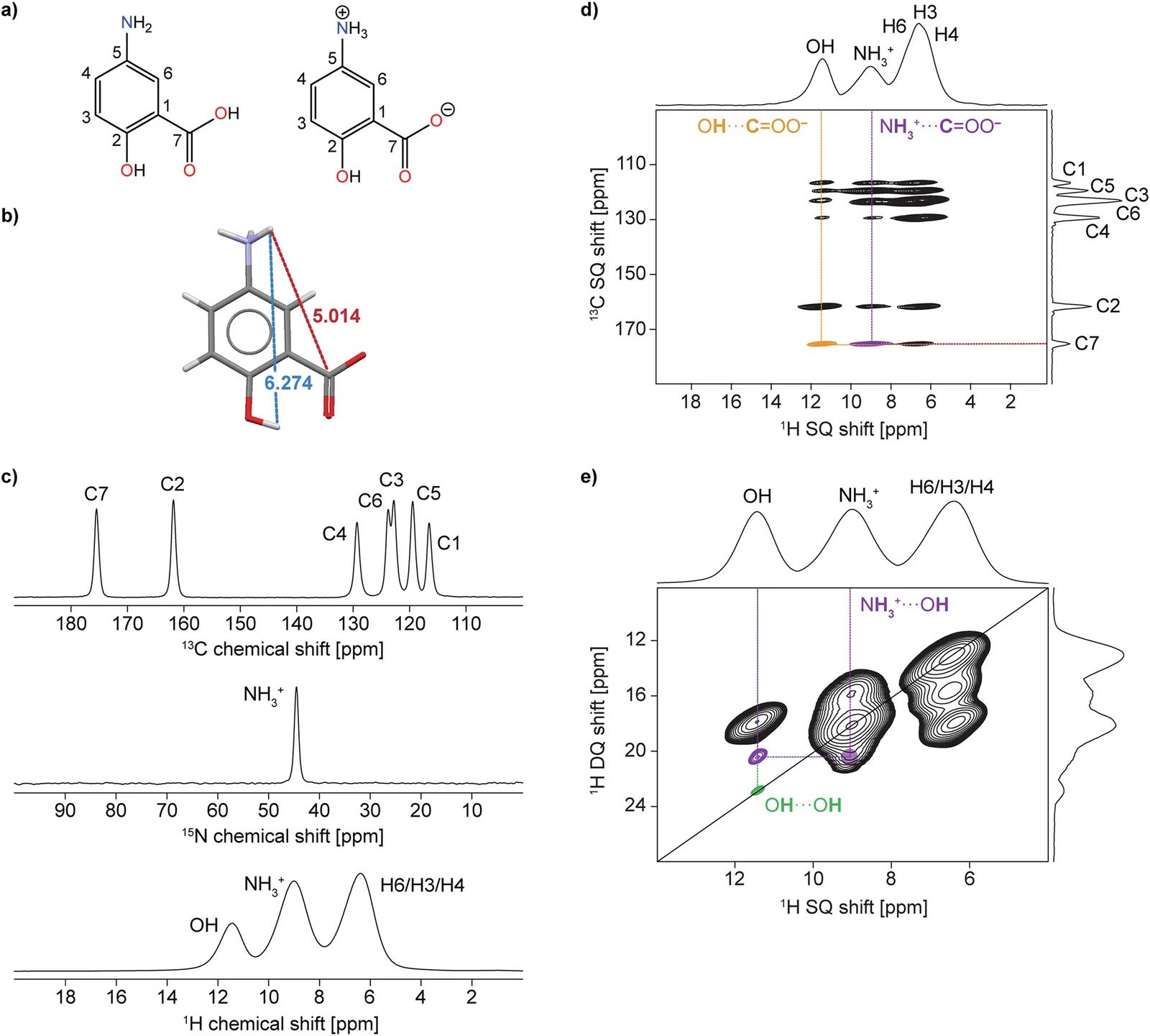
SSNMR spectra of 5-ASA. **a** Chemical structures with atom labelling of neutral and zwitterionic 5-ASA. **b** Intramolecular distances in 5-ASA molecular structure, built using Mogul, between the amine protons and the carboxylate carbon (red) and the hydroxyl proton (blue). **c** ^13^C (150.91 MHz) and ^15^N (60.82 MHz) CPMAS and ^1^H (600.17 MHz) MAS spectra of 5-ASA acquired at room temperature at a spinning speed of 20, 9, and 70 kHz, respectively. **d** Long-range ^1^H-^13^C Double-CP spectrum (ct2 = 2 ms) of 5-ASA acquired at room temperature at a spinning speed of 70 kHz. The correlations between the OH and NH_3_^+^ protons with the carboxylate carbon are highlighted in orange and purple, respectively. **e** ^1^H DQ/^1^H SQ MAS spectrum of 5-ASA acquired at room temperature at a spinning speed of 70 kHz. The correlation between the OH and NH_3_^+^ protons is highlighted in purple, while the autocorrelation OH···OH in green.

**Fig. 2:**
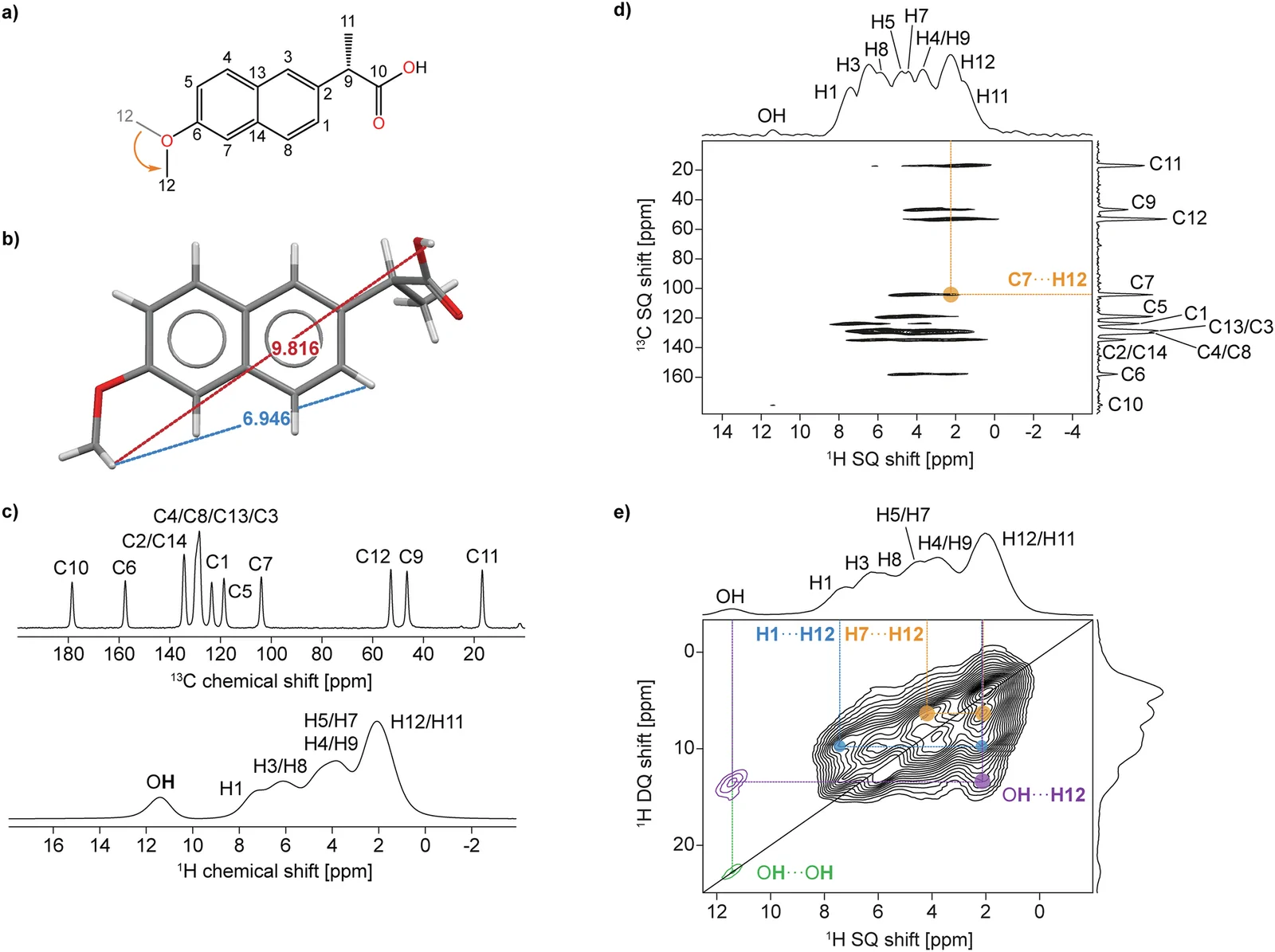
SSNMR spectra of S-NAP. **a** Chemical structure with atom labelling of S-NAP. The conformation observed by SSNMR is shown with the orange arrow. **b** Intramolecular distances in S-NAP molecular structure, built using Mogul, between the methoxy protons and the aromatic H1 (blue) and the hydroxyl proton (red). **c** ^13^C (150.91 MHz) CPMAS and ^1^H (600.17 MHz) MAS spectra of S-NAP acquired at room temperature at a spinning speed of 20 and 70 kHz, respectively. **d** Long-range ^1^H-^13^C Double-CP spectrum (ct2 = 2 ms) of S-NAP acquired at room temperature at a spinning speed of 70 kHz. The correlation between C7 and the methoxy protons is highlighted in orange. **e** ^1^H DQ/^1^H SQ MAS spectrum of S-NAP acquired at room temperature at a spinning speed of 70 kHz. The correlations of the methoxy protons (H12) with H7, H1, and OH are highlighted in orange, blue, and purple, respectively. In green is shown the OH···OH autocorrelation.

To develop and test the Global-PDF-Fit/SSNMR approach, two model compounds were selected, differing slightly in molecular size, i.e., number of atoms in the molecule, and functional groups, with previously published crystal structures. The selected compounds are 5-aminosalicylic acid (5-ASA, Fig. 1a) and S-naproxen (S-NAP, Fig. 2a), which exhibit a calculated crystallite size of ∼50 and ∼55 nm, respectively. All details regarding materials, powder X-ray diffraction data (from both laboratory instruments and synchrotron) analysis and PDF generation are reported in Supplementary Sections 1 and 2, and Supplementary Figs. 1–8.

### SSNMR analysis

The model compounds were thoroughly characterized using both 1D and 2D SSNMR experiments, including ^13^C and ^15^N CPMAS, ^1^H MAS, ^1^H-^13^C Double-CP, ^1^H-^14^N T-HMQC, and ^1^H DQ/^1^H SQ MAS experiments. All experimental parameters are reported in Supplementary Section 3 and Supplementary Table 1. The experimental ^1^H, ^13^C, and ^15^N chemical shifts for 5-ASA and the experimental ^1^H and ^13^C ones for S-NAP, together with their corresponding assignments, are summarized in Supplementary Tables 2 and 4, respectively.

#### 5-ASA

The 1D ^1^H MAS, ^13^C and ^15^N CPMAS, and 2D long-range ^1^H-^13^C Double-CP and ^1^H DQ/^1^H SQ MAS SSNMR spectra of 5-ASA, as well as its chemical structure with the atom labelling here used, are reported in Fig. 1, while the ^1^H-^14^N T-HMQC and the short-range ^1^H-^13^C Double-CP spectra are reported in Supplementary Figs. 9 and 10. Analysis of the number of resonances in the ^13^C and ^15^N CPMAS SSNMR spectra (Fig. 1c) indicates the presence of one independent molecule of 5-ASA in the unit cell (*Z’* = 1). Moreover, the chemical shift of the amine ^15^N signal at 44.6 ppm suggests that 5-ASA exists in the zwitterionic form in the solid state, i.e., characterized by the NH_3_^+^ group^55,56^. This was further confirmed through the integration of the signals in the quantitative ^1^H MAS spectrum, as resumed in Supplementary Table 3 (Fig. 1c).

The O**H**···**C**OO^–^ correlation detected in the long-range ^1^H-^13^C Double-CP spectrum (Fig. 1d), together with the relatively high chemical shift of the O**H** signal in the ^1^H MAS spectrum (δ_iso_ = 11.4 ppm, Fig. 1c), suggests the presence of an intramolecular hydrogen bond between them, likely with an *S*(6) motif. Analysis of the same motif in neutron diffraction structures present in the Cambridge Structural Database (CSD) (Supplementary Section 4.1 and Supplementary Table 5) reveals typical O···O distances of ~2.6 Å and bond angles around 145–150°. According to Jeffrey^57^, hydrogen bonds with acceptor–donor distances (*r*_AD_) greater than 2.5 Å and bond angles between 130° and 175° are typical of moderate hydrogen bonds in agreement with the ^1^H chemical shift of the OH signal at 11.4 ppm^38^. Moreover, the N**H**_3_^+^···**C**OO^–^ correlation in the long-range ^1^H-^13^C Double-CP spectrum (Fig. 1d), together with the O**H**···N**H**_3_^+^ correlation observed in the ^1^H DQ/^1^H SQ MAS spectrum (δ_DQ_ ~ 20.3 ppm, Fig. 1e), indicates a close intermolecular proximity between these groups. An intramolecular origin of these correlations can be ruled out based on their intramolecular distances. Indeed, due to the planarity of the molecule, the amine protons are located more than 5 Å from the carboxylic carbon and over 6 Å from the hydroxyl proton (Fig. 1b). These observations point to the formation of a hydrogen bond interaction involving the NH_3_^+^ moiety of one 5-ASA molecule and the COO^–^ of a neighbouring one. Finally, the presence of the O**H**···O**H** autocorrelation in the ^1^H DQ/^1^H SQ MAS spectrum (δ_DQ_ ~ 22.8 ppm, Fig. 1e) suggests a spatial proximity, within 3.5–4 Å, between hydroxyl groups belonging to two different molecules.

#### S-NAP

The 1D ^1^H MAS and ^13^C CPMAS, and 2D long-range ^1^H-^13^C Double-CP and ^1^H DQ/^1^H SQ MAS SSNMR spectra of S-NAP, along with its chemical structure and the atom labelling scheme used herein, are shown in Fig. 2. The short-range ^1^H-^13^C Double-CP is reported in Supplementary Fig. 11. As previously outlined for 5-ASA, the analysis of the number of signals in the ^13^C CPMAS SSNMR spectrum confirms the presence of one independent molecule of S-NAP in the unit cell (*Z*′ = 1). (Fig. 2c)

The correlation C7**H**···OC**H**_3_ (δ_DQ_ = 6.5 ppm) observed in the ^1^H DQ/^1^H SQ MAS spectrum (Fig. 2e), along with the correlation between C7 and the methoxy protons detected in the long-range ^1^H-^13^C Double-CP spectrum (Fig. 2d), suggests that the methoxy moiety is oriented toward C7 rather than C5. The OH of the carboxylic group participates in a moderate intermolecular hydrogen bond, as evidenced by the ^1^H chemical shift at 11.5 ppm (Fig. 2c). This, together with the weak O**H**···O**H** autocorrelation observed in the ^1^H DQ/^1^H SQ MAS spectrum (δ_DQ_ ~ 23.0 ppm, Fig. 2e) supports the formation of a chain-like hydrogen-bond motif rather than a homodimer. Indeed, an analysis of the intermolecular H–H distances in COOH···HOOC homodimers, performed with ConQuest (CSD)^58^, reveals that an average intermolecular H–H distance of ~2.3 Å is typically found in such motifs (Supplementary Section 4.2 and Supplementary Fig. 12). Such short contacts should give rise to O**H**···O**H** autocorrelations of higher intensity than what observed for S-NAP. Moreover, carboxylic homodimers generally involve strong hydrogen bonds, typically showing a carboxylate-like character, which would also result in a shift of the carboxylic ^13^C resonance to higher frequencies than the one observed (δ_iso_ = 178.6 ppm, Fig. 1c)^59^.

Further insights into the molecular spatial arrangement come from the correlations in the ^1^H DQ/^1^H SQ MAS spectrum between the hydroxyl and methoxy protons (δ_DQ_ ~ 13.7 ppm), as well as between the methoxy protons and the aromatic H1 (δ_DQ_ ~ 9.6 ppm), suggesting intermolecular proximities shorter than 3.5–4 Å between these moieties. Assuming a nearly planar conformation of the molecule, it can be undoubtedly ruled out the intramolecular origin of these correlations, given their intramolecular OC**H**_3_···**H1** and OC**H**_3_···O**H** distances of ~6.9 Å and ~9.8 Å, respectively (Fig. 2b). This last finding supports an anti-parallel arrangement of the S-NAP molecules.

### Application of the Global-PDF-Fit/SSNMR approach

The first important contribution of SSNMR lies in the definition of the molecular input for the structure generation. The zwitterionic character of 5-ASA and the presence of the OH···COOH intramolecular hydrogen bond were considered as structural restraints during the generation of the molecular input. For S-NAP, only the orientation of the methoxy moiety was considered. Although FIDEL-GO allows the inclusion of intramolecular degrees of freedom, such as torsion angles or rotations of flexible molecular fragments, to sample the conformational space, these were not refined in the present work. During method development, the molecular conformations observed in the experimental crystal structure of both 5-ASA (SAQJAV03^60^) and S-NAP (COYRUD13^61^) were adopted, and the molecules were treated as rigid throughout the structure-generation, PDF-comparison, and PDF-fit steps in order to save computational time.

Random structure generation runs were performed in the most frequent space groups for organic molecules according to the survey published by the CCDC in 2025^62,63^. For 5-ASA, calculations were performed in the *P*2_1_/*c* (*Z* = 4), *P*1 (*Z* = 1), $P\bar{1}$ (Z = 2), *P*2_1_ (*Z* = 2), and *P*2_1_2_1_2_1_ (*Z* = 4) space groups. In the case of S-NAP, the exclusive presence of the *S* enantiomer in the asymmetric unit ruled out all enantiogenic (*i.e*., non-Sohncke) space groups, and calculations were therefore performed in *P*2_1_ (*Z* = 2), *P*1 (*Z* = 1), and *P*2_1_2_1_2_1_ (*Z* = 4)^62^. For both 5-ASA and S-NAP, only *Z’* = 1 was considered, as determined by SSNMR analysis. During the process of structure generation, the lattice parameters ranges were automatically chosen depending on the size of the investigated molecule and the space group in which the calculations are performed (e.g., 3–40 Å for a molecule with a maximal length of 10 Å in the space group *P*2_1_2_1_2_1_ with *Z* = 4). More details about the lattice parameters ranges used for structure generation are given in the Supplementary Information, Section 5.1. Random starting values for the molecular orientation and position were used.

Multiple structure generation runs were performed for both compounds, leading to the generation of millions of structural models. Among these, only the structures that did not exhibit any kind of molecular overlap were considered, leading to the actual generation and preliminary evaluation of 312,852 and 176,867 crystal structures for 5-ASA and S-NAP, respectively (Supplementary Table 8). The simulated PDFs of these structures were compared with the experimental one using cross-correlation function and then ranked by similarity^53^. The structures were selected based on a minimum value of the similarity measure, *S*_12_, between the simulated and experimental PDF (2^nd^ selection, Scheme 1b). The used similarity thresholds are reported in Supplementary Table 9. At this point, only the most promising structures were retained for the next steps, resulting in 2241 structure candidates for 5-ASA, and 2418 for S-NAP. The remained structures were then subjected to a multi-step fit to the experimental PDF using TOPAS and ranked based on their ${R}_{{wp}}^{{PDF}}$ values. Further details on the PDF fit are provided in Supplementary Table 10.

After the PDF fit, SSNMR-derived intermolecular H–H proximities were used as constraints to filter out all inherently wrong structures. For both compounds, all intermolecular H–H contacts were calculated and analysed using an automated procedure implemented in in-house developed Python scripts. The same contacts analysis can be performed also using Mercury. To test the performance of the filtering step, the intermolecular H–H contacts were evaluated over 5 different ranges: 0–3.50 Å, 0–3.75 Å, 0–4.00 Å, 0–4.25 Å, and 0–4.50 Å. Only the structures exhibiting the desired intermolecular H–H contacts detected in ^1^H DQ/^1^H SQ MAS spectra were retained. In the case of 5-ASA, structures lacking either the OH···OH and OH···NH_3_^+^ intermolecular contacts within the considered range were discarded, whereas for S-NAP those lacking OH···OH, OCH_3_···H1, and OCH_3_···OH intermolecular contacts were eliminated. The results of the SSNMR-based filtering step are reported in Table 1.

**Table 1:** Percentages (%) of 5-ASA and S-NAP structures erased after the SSNMR-based filtering step using different ranges of intermolecular H–H distances

	Percentage of erased structures after filtering (%)				
	0–3.50 Å	0–3.75 Å	0–4.00 Å	0–4.25 Å	0–4.50 Å
**5-ASA**	88.2	76.2	57.1	42.8	32.5
**S-NAP**	98.6	97.3	95.1	89.8	83.5

As shown in Table 1, the outcomes of the SSNMR-based filtering step strongly depend on the range used for the proximity calculation. The best results in terms of percentage of erased structures are obtained using the 0–3.50 Å or 0–3.75 Å ranges. However, correlations can still be observed in ^1^H DQ/^1^H SQ MAS spectra for contacts up to ∼4.00 Å. Although higher upper limits, such as 4.25 and 4.50 Å, result more conservative, they lead to a noticeable reduction in the filtering efficiency. Based on this consideration, and in accordance with the generally reported maximum distances detectable by ^1^H DQ/^1^H SQ MAS SSNMR experiments (i.e., 3.50–4.00 Å)^38^, the 0–4.00 Å range was selected as the best compromise between reliability and efficiency. However, it should be noted that the optimal cutoff may vary case-by-case and relies on careful interpretation of the SSNMR spectra. In cases where the SSNMR data are uncertain, the use of slightly larger upper limits may represent a safer and more conservative approach. Using the 0–4.00 Å range, for 5-ASA the filtering step led to the exclusion of 57.1% of the candidate structures, resulting in 962 remaining crystal structures out of the initial 2241 (Supplementary Table 11). For S-NAP, instead, the impact of the SSNMR-based filtering step is remarkably higher, enabling to remove 95.1% of the structural models, resulting in 118 out of 2418 structures (Supplementary Table 12). It is worth noting that this filtering step allows to remove also top-${R}_{{wp}}^{{PDF}}$-ranked structural models, e.g., the 2^nd^, 3^rd^, or 4^th^ best ones. As an example, the impact of the filtering on the top 40 structures (for both 5-ASA and S-NAP), ranked by ${R}_{{wp}}^{{PDF}}$ value, is illustrated in Supplementary Tables 13 and 14, highlighting the filtering power of the SSNMR-derived constraints.

Although the SSNMR-filtering step for 5-ASA was already highly effective, the better performance observed for S-NAP can be mainly attributed to the different molecular size, *i.e*., number of atoms and length of the molecule. This affects both the number and the effectiveness of the intermolecular proximities that can be used as constraints during the filtering step. Larger molecules facilitate the unambiguous identification of purely intermolecular correlations in 2D SSNMR spectra. Indeed, in larger molecules, many intramolecular distances are too long to contribute significantly to the detected correlations, which simplifies the assignment of intermolecular contacts. Moreover, changes in the orientation and/or position of a small molecule in the unit cell produce only subtle changes in the intermolecular atom–atom distances. By contrast, for a larger molecule, different positions and orientations in the unit cell can lead to very different intermolecular distances, making the constraints more discriminating and resulting in a more selective filtering process.

Finally, DFT-D geometry optimisations were carried out, starting from the best 10 candidate structures for both 5-ASA and S-NAP, i.e., those with the lowest ${R}_{{wp}}^{{PDF}}$ values after the SSNMR-based filtering step (Supplementary Tables 15, 16). Crystallographic information files for all optimised crystal structures are provided in the Supplementary Information. SSNMR chemical shifts were computed only for the structures with relative energies in the range 0–10 kJ/mol (Table 2, Supplementary Figs. 13, 14).

**Table 2:** Relative energies for the optimised structural models of 5-ASA and S-NAP

5-ASA			
Structure	ΔE (kJ/mol)	^13^C RMSE (ppm)	^1^H RMSE (ppm)
R1k_5348	0.00	1.0	0.3
R1f_6112	17.31	/	/
R3a_4273	41.36	/	/
R3a_8396	41.93	/	/
R3a_9624	51.90	/	/
R3a_9385	64.71	/	/
R1i_48369	70.76	/	/
R3a_9334	80.66	/	/
R3a_12919	101.60	/	/
R3a_12130	150.58	/	/
**S-NAP**			
**Structure**	**ΔE (kJ/mol)**	^**13**^**C RMSE (ppm)**	^**1**^**H RMSE (ppm)**
R1b_1041	0.00	1.1	0.5
R1h_8277*	0.02	1.1	0.5
R1d_2664**	5.86	1.5	0.6
R1f_687	9.24	1.4	0.3
R2d_1647	25.41	/	/
R2a_12	35.93	/	/
R2a_382	44.46	/	/
R2a_413	54.14	/	/
R2c_405	57.55	/	/
R1g_2473	62.25	/	/

^1^H and ^13^C chemical shift RMSEs were calculated only for the structures with relative energies in the range 0–10 kJ/mol.

^*^R1h_8277 is virtually identical to R1b_1041 post-DFT-D optimisation.

**R1d_2664 exhibits the same molecular arrangement of R1b_1041 post-DFT-D optimisation, with just small deviations of the lattice parameters and a slight molecular distortion.

The final selection of the correct crystal structures was made by jointly evaluating the relative energy of the candidate structures and the ^1^H and ^13^C chemical shifts RMSEs. In the case of 5-ASA, after DFT-D optimisation, only one structure was found in the range 0–10 kJ/mol, i.e., R1k_5348. Thus, SSNMR calculations were performed only for this structure (Supplementary Table 2), resulting in excellent ^13^C and ^1^H SSNMR RMSEs of 1.0 and 0.3 ppm^64^, respectively, suggesting that R1k_5348 is the correct structural model. Concerning S-NAP, after DFT-D optimisation, the first three best candidate structures exhibit the same molecular arrangement, with the first two (i.e., R1b_1041 and R1h_8277) being identical, and with structure R1d_2664 exhibiting only slight deviation in the molecular orientation. An overlay of the three structures is shown in Supplementary Fig. 15. On the base of the energy differences, the ^13^C and ^1^H RMSEs, and the equivalence of three of the optimised structures, the candidate structure R1b_1041 was selected as the correct one. The calculated ^13^C and ^1^H SSNMR chemical shifts of structure R1b_1041 of S-NAP are listed in the Supplementary Table 4.

**Fig. 3:**
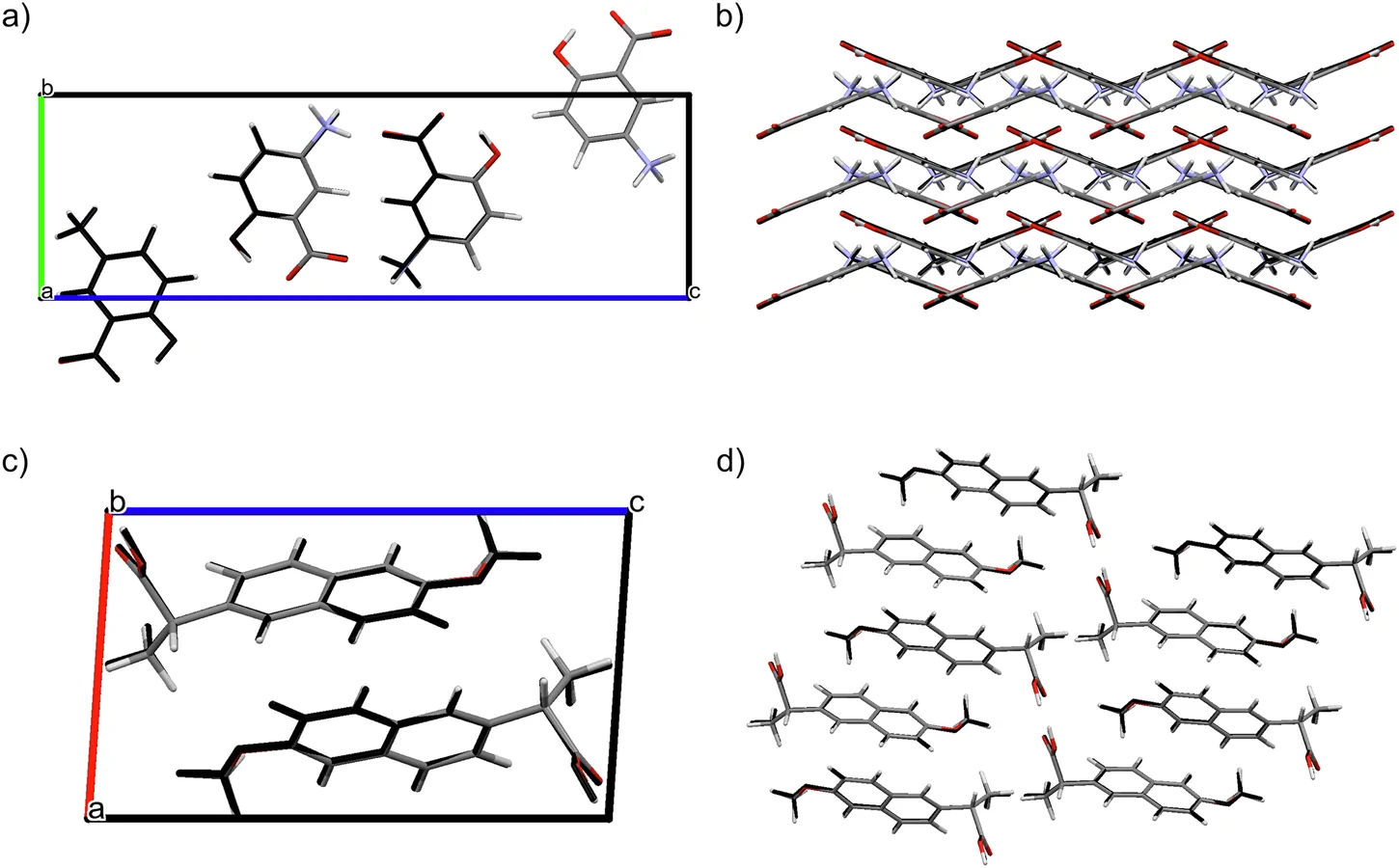
Overlay of the candidate structures of 5-ASA and S-NAP with the experimental ones. **a**, **b** Overlay of structure R1k_5348 (standard colours) with the experimental structure of 5-ASA (SAQJAV03, black). **c**, **d** Overlay of structure R1b_1041 (standard colours) with the experimental structure of S-NAP (COYRUD13, black).

For both 5-ASA and S-NAP, the selected crystal structure perfectly matches the experimental one. The PDF fits of structures R1k_5348 and R1b_1041 are shown in Supplementary Figs. 16, 17, respectively. Structural comparisons with the experimental structures using Mercury^65^ show a root-mean square deviation (RMSD) as low as 0.06 Å for structure R1k_5348 of 5-ASA (Fig. 3a, b) and 0.05 Å for structure R1b_1041 of S-NAP (Fig. 3c, d). Powder pattern comparisons further confirm the correctness of the selected structures; indeed, the simulated powder patterns of the determined structures closely reproduce the experimental data (Supplementary Figs. 18, 19).

## Conclusions

In the present work, a combined method for crystal structure determination of small organic molecules, namely the Global-PDF-Fit/SSNMR approach, was developed by integrating SSNMR and PDF analysis. The method proved to be a successful and powerful strategy for structure determination, as demonstrated for two model compounds, 5-aminosalicylic acid and S-naproxen.

The complementary nature of SSNMR and PDF provides access to both local and intermediate-range structural information, enabling effective filtering and selection of the candidate structures. The presented workflow is highly versatile and can be directly transferred to the standard FIDEL-GO procedure using powder diffraction data, without modification of the underlying strategy. This flexibility highlights the general applicability of the method for integrating experimental constraints into structure refinement and validation protocols, underscoring its broader relevance for structural studies beyond the specific system investigated here. In particular, the proposed structure-filtering strategy can be widely applied to all cases where thousands or millions of candidate structures need to be pre-selected, e.g., in Crystal Structure Prediction (CSP), dramatically reducing the time required for structure selection. Furthermore, increasing molecular size, which is usually one of the main issues of CSP procedures, due to very long and computationally expensive selection process, represents a distinct advantage for the present methodology. Larger molecules exhibit a richer network of SSNMR-detectable intermolecular proximities, many of which are directly accessible through ^1^H DQ/^1^H SQ MAS SSNMR experiments. These additional constraints enhance sensitivity to packing variations and further strengthen the discriminatory power of the combined Global-PDF-Fit/SSNMR approach for complex molecular solids.

Although demonstrated on pharmaceutical samples with crystallite sizes of ∼50–55 nm, the method can be extended to other classes of compounds, such as metal complexes and metal-organic frameworks (MOFs), and to significantly more nanocrystalline materials, with crystallite sizes down to at least 10–20 nm, for which single-crystal and standard powder diffraction methods are not feasible due to the pronounced peak broadening and overlap. In such cases, the synergistic use of SSNMR and PDF can provide a robust pathway for obtaining reliable structural models, as both techniques can provide detailed local-structure information even in nanocrystalline (with small crystallite size, below 40 nm) and amorphous systems. Moreover, the structure filtering based on SSNMR-detected intermolecular contacts, and the comparison of computed and experimental chemical shifts, provide alternative selection criteria to the ${R}_{{wp}}^{{PDF}}$ values used to rank the generated structures, increasing the reliability of the selected structure, especially in the case of samples with very low crystallite size. This underscores the advantage of integrating diffraction-based approaches with SSNMR, as realized in the Global-PDF-Fit/SSNMR workflow.

## Methods

### Materials

5-aminosalicylic acid (5-ASA) and S-naproxen (S-NAP) were purchased from Thermo Scientific and BLDpharm, respectively, and used without further purification.

### Powder X-ray diffraction (PXRD)

X-ray powder patterns were acquired on a Stoe Stadi-P diffractometer equipped with a primary Ge(111) monochromator and a Mythen detector, using Cu-K_α1_ radiation (λ = 1.54506 Å). Measurements were performed over a 2θ range of 5–50°, using 0.7 mm glass capillaries. Capillaries were spun during measurements.

### Pair Distribution Function (PDF) analysis

Synchrotron data were collected at the PETRA III P02.1 beamline (DESY)^66^, using a wavelength of 0.207377 Å and 1 mm glass capillaries. The PDFs, G(*r*), were obtained from the total-scattering data by background correction, normalization, and Fourier transformation using PDFgetX3^67^.

### Solid-state NMR (SSNMR)

1D and 2D SSNMR spectra (^13^C and ^15^N CPMAS, ^1^H MAS, ^1^H-^13^C Double-CP, ^1^H-^14^N T-HMQC, and ^1^H DQ/^1^H SQ MAS) were acquired under MAS conditions at room temperature on a Jeol ECZR 600 instrument, operating at 600.17, 150.91, and 60.82 MHz for ^1^H, ^13^C, and ^15^N nuclei, respectively. ^13^C and ^15^N CPMAS spectra were acquired using a ramped cross-polarization pulse sequence and a two-pulse phase modulation (TPPM) decoupling scheme for ^13^C and a SPINAL64 decoupling scheme for ^15^N. The ^13^C and ^15^N chemical shift scales were calibrated through the methylenic signal of the external standard adamantane (δ(^13^C) = 38.48 ppm with reference to TMS) and the NH_3_^+^ signal of external standard α-glycine (δ(^15^N) = 33.4 ppm with reference to liquid NH_3_), respectively. For the ^1^H MAS, ^1^H-^13^C Double-CP MAS, ^1^H-^14^N T-HMQC, and ^1^H DQ/^1^H SQ MAS spectra, the ^1^H radio frequency (rf) field strength was 285.7 kHz at a *π*/2 pulse length of 0.875 μs. The ^1^H MAS spectra were acquired using a DEPTH^68^ pulse sequence with optimized recycle delays of 5*T_1_^1^H to ensure a quantitative response. The ^1^H-^13^C Double-CP MAS experiments were performed using second contact times of 0.2 ms and 2 ms to investigate short- and long-range correlations, respectively. The ^1^H DQ/^1^H SQ MAS experiments were performed with the back-to-back (BABA) recoupling pulse sequence. The ^1^H chemical shift scale was calibrated *via* the methylenic signal of the external standard alanine (δ(^1^H) = 1.5 ppm with reference to TMS).

### Cambridge Structural Database (CSD) analysis

Motif searches were performed using ConQuest within the Cambridge Structural Database (CSD).

### FIDEL parameters for structure comparison with cross-correlation function

After removing the structures exhibiting atomic overlap, the remaining hundreds of thousands of structural models (Supplementary Table 8) were compared to the experimental PDF using the similarity measure *S*_12_ in an automated multi-step process, which includes ranking according to the similarity to PDF using cross-correlation functions, clustering and removal of duplicates. A detailed description of the comparison of PDFs using cross-correlation functions is given by Habermehl et al.^14,53,54,69^. The similarity measure *S*_12_ can take values from 0 to 1, where a value of 1 corresponds to identical PDFs. Even if an *S*_12_ threshold of 1 could theoretically be used, the main aim of the PDFs comparison step is to rule out unreasonable structural models rather than selecting the correct one. For this reason, *S*_12_ thresholds are usually set to values close to 1, e.g., 0.98, which allows for the selection of promising structural models, which will be then fitted to the PDF, while maintaining reasonable computational time. Different *S*_12_ thresholds can be used in different runs, based on the computational resources and time available. The *S*_12_ thresholds used for the PDFs comparison of 5-ASA and S-NAP are reported in Supplementary Table 9.

### DFT-D optimisation and NMR calculations

Crystal structures were optimized at DFT-D level with Quantum Espresso (QE, v. 6.4.1)^70^, employing the projector augmented wave (PAW) approach, with the non-local vdW-df2 method^71^ and the B86r functional^72^ with the SSSP set of pseudopotentials^73^. During the optimisation the unit cell parameters were fixed and only the atomic coordinates were refined. An energy cut-off of 60 Ry was used. Starting from the optimized structures with fixed lattice parameters, NMR calculations were performed using the GIPAW^74^ and the PBE pseudopotentials from PS Library 1.0.0^75^ with an energy cut-off of 80 Ry, following the methodology previously described^76,77^. The theoretical absolute isotropic magnetic shielding (σ_iso_) values were converted into isotropic chemical shifts (δ_iso_) applying the linear regression method^78,79^.

### Structure analysis

Structural visualization and comparison were carried out using Mercury. Structural similarity was evaluated using Cartesian root-mean square deviation (RMSD) *via* the Crystal Packing Similarity tool.

Full experimental and computational details are provided in the Supplementary Information.

## Supplementary information

Supplementary Information

## Data Availability

The authors declare that all data supporting the findings of this study are available within the paper and its Supplementary Information files, except for the plots comparing the simulated and experimental PDFs for all generated structures, which are available from the corresponding author upon reasonable request. The raw SSNMR data, the synchrotron diffraction data, the generated PDFs, the top 10 ranked crystal structures, pre and post DFT-D optimisation, and the calculated NMR shielding values are also deposited in Zenodo (https://doi.org/10.5281/zenodo.21685947). Furthermore, the raw SSNMR data are available also through EXPANDO. All deposited SSNMR spectra of the desired compounds are  easily accessible *via* a search by name on the search page of the database.

## Code availability

The in-house developed Python scripts used for the calculation and analysis of intermolecular H–H contacts are available from the corresponding author upon reasonable request.
