# Supplementary material for: Pair distribution function “feat.” solid-state NMR in a combined approach for structure determination of organic molecules
Source: Commun Chem. 2026 Oct 6;9:314. doi: 10.1038/s42004-026-02233-x (PMC13642911; doi:10.1038/s42004-026-02233-x)
Supplement: Supplementary file 1 [file 42004_2026_2233_MOESM1_ESM.pdf]

Pair distribution function "feat." solid-state NMR in a combined approach for structure determination of organic molecules

Supplementary Information

Chiara Rosso,[1 ‡] Toms Rekis,[2] Michele R. Chierotti,[1] Federica Bravetti[2 ‡ *]

[1] Department of Chemistry and NIS Center, University of Turin, Via P. Giuria 7, 10125, Turin, Italy

[2] Institute of Inorganic and Analytical Chemistry, Goethe University, Max-von-Laue-Str. 7, 60438, Frankfurt am Main, Germany

* Corresponding author: Bravetti@chemie.uni-frankfurt.de

‡ These authors contributed equally

Table of contents

Description	Page number
**Figure 1.** Powder patterns from experimental powder (black) and simulated from crystallographic data of the crystal structure SAQJAV03 (green) of 5-ASA in a 2θ range of 5–50°.	5
**Figure 2.** Powder patterns from experimental powder (black) and simulated from crystallographic data of the crystal structure COYRUD13 (green) of S-NAP in a 2θ range of 5–50°.	6
**Figure 3.** Synchrotron powder pattern of 5-ASA in a 2θ range of 0.5–63.5°.	6
**Figure 4.** Synchrotron powder pattern of S-NAP in a 2θ range of 0.5–63.5°.	7
**Figure 5.** Experimental PDF curve of 5-ASA (Q_{max} = 20.0 $Å^{-1}$).	7
**Figure 6.** Experimental PDF curve of S-NAP (Q_{max} = 20.0 $Å^{-1}$).	8
**Figure 7.** Overlay of the PDFs of three different measurements of the same sample of 5-ASA (Q_{max} = 20.0 $Å^{-1}$).	8
**Figure 8.** Overlay of the PDFs of two different measurements of the same sample S-NAP (Q_{max} = 20.0 $Å^{-1}$).	9
**Table 1.** ^{1}H MAS, ^{13}C and ^{15}N CPMAS, ^{1}H-^{13}C short- and long-range Double-CP MAS, ^{1}H-^{14}N T-HMQC, and ^{1}H DQ/^{1}H SQ MAS experimental parameters for 5-ASA and S-NAP.	10
**Figure 9.** ^{1}H-^{14}N T-HMQC spectrum of 5-ASA acquired at room temperature at a spinning speed of 70 kHz. The correlation enabling the assignment of the protons of the protonated amine moiety is highlighted with dashed red lines.	11
**Figure 10.** Short-range ^{1}H-^{13}C Double-CP spectrum of 5-ASA acquired at room temperature at a spinning speed of 70 kHz. The correlations between the CH carbons and their covalently bound protons are highlighted with dashed red lines.	11
**Table 2.** Experimental and computed ^{1}H, ^{13}C, and ^{15}N chemical shifts and corresponding assignment for 5-ASA.	12
**Table 3.** General assignment and corresponding integrals of the ^{1}H signals of the quantitative ^{1}H MAS spectrum of 5-ASA.	12
**Figure 11.** Short-range ^{1}H-^{13}C Double-CP spectrum of S-NAP acquired at room temperature at a spinning speed of 70 kHz. The correlations between the CH carbons and their covalently bound protons are highlighted with dashed red lines.	12

**Table 4.** Experimental and computed ^{1}H and ^{13}C chemical shifts and corresponding assignment for S-NAP.	13
**Table 5.** Results of the motif search performed with ConQuest (CSD version 5.45).	14
**Figure 12.** Histogram plot of the intermolecular H–H distance for the 31 entries of the ConQuest search.	14
**Table 6.** Lattice parameters ranges used during structure generation of 5-ASA.	15
**Table 7.** Lattice parameters ranges used during structure generation of S-NAP.	17
**Table 8.** Number of structures generated and compared to the experimental PDF using a cross-correlation function for 5-ASA and S-NAP. The total number of generated structures, before checking for atomic overlap, is even higher (~10^6). Plots of the comparison of the simulated and experimental PDFs for all the structures are available on request.	18
**Table 9.** FIDEL calculation parameters for structure generation of 5-ASA and S-NAP.	19
**Table 10.** FIDEL-TOPAS PDF-fit algorithm used for calculations of both 5-ASA and S-NAP. Legend: x = gets refined; n.a. = not applied; fixed = no refinement, value fixed to result from previous refinement; fixed to V = no refinement, value fixed to given value (system/sample dependent)*; macro pdf_mode 2 = "for simulated annealing with z-Matrix and B_{iso}-intra/B_{iso}-inter; macro pdf_mode 0 = "for refinement with Bintra/Binter (default)".	20
**Table 11.** Results of the structure filtering using the H–H (OH···OH and OH···NH_3^+) intermolecular contacts as constraints for 5-ASA. The label "Starting structures" refers to the structures that exhibited a good agreement after the comparison of the simulated and experimental PDFs using a cross-correlation function, and not to the total number of generated structures.	21
**Table 12.** Results of the structure filtering using the H-H (OH···OH, OCH_3···H1, and OCH_3···OH) intermolecular contacts as constraints for S-NAP. The label "Starting structures" refers to the structures that exhibited a good agreement after the comparison of the simulated and experimental PDFs using a cross-correlation function, and not to the total number of generated structures.	22
**Table 13.** Result of the structures filtering on the best 40 structures of 5-ASA, ranked according to the R_{wp}^{PDF}. Structures highlighted in red were discarded according to SSNMR constrains.	23
**Table 14.** Result of the structures filtering on the best 40 structures of S-NAP, ranked according to the R_{wp}^{PDF}. Structures highlighted in red were discarded according to SSNMR constrains.	24

**Table 15.** Results of the DFT-D optimisation with fixed cell parameters for the best 10 structures of 5-ASA. The structures are ranked according to their relative lattice energy.	25
**Table 16.** Results of the DFT-D optimisation with fixed cell parameters for the best 10 structures of S-NAP. The structures are ranked according to their relative lattice energy.	25
**Figure 13.** Plots of the calculated *vs* experimental ^{13}C and ^{1}H chemical shifts for the best candidate structure for 5-ASA, R1k_5348.	26
**Figure 14.** Plots of the calculated *vs* experimental ^{13}C and ^{1}H chemical shifts for the best four candidate structures for S-NAP.	27
**Figure 15.** Overlay of structure candidate R1b_1041 (standard colors) with a) structure candidate R1h_8277 (in green) and b) structure candidate R1d_2664 (in green). The overlay was done with the crystal packing similarity tool of Mercury, considering a cluster of 30 molecules and a tolerance of 20%. The resulting RMSCD values are 0.033 Å and 0.633 Å for R1h_8277 and R1d_2664, respectively.	28
**Figure 16.** PDF-fit plot of structure R1k_5348 (5-ASA). Experimental PDF in black, calculated PDF in red, and difference curve in blue.	29
**Figure 17.** PDF-fit plot of structure R1b_1041 (S-NAP). Experimental PDF in black, calculated PDF in red, and difference curve in blue.	29
**Figure 18.** Comparison of the experimental powder pattern of 5-ASA (Exp 5-ASA) with the simulated powder pattern of the candidate structure R1k_5348 (Sim R1k_5348).	30
**Figure 19.** Comparison of the experimental powder pattern of S-NAP (Exp S-NAP) with the simulated powder pattern of the candidate structure R1b_1041 (Sim R1b_1041).	30
**References**	31

1. Materials

5-ASA (5-aminosalicylic acid ($C_7H_7NO_3$), 99%), was purchased from Thermo Scientific, while S-NAP (S-naproxen, (2*S*)-2-(6-methoxynaphthalen-2-yl)propanoic acid ($C_{14}H_{14}O_3$), 99.91%) was purchased from BLDpharm. Both samples were analysed as such without further purification steps.

2. Powder X-ray diffraction

2.1 Powder diffraction for phase analysis

X-ray powder patterns of the powdered samples were acquired on a Stoe Stadi-P diffractometer, equipped with a primary Ge(111) monochromator and with a Mythen detector, using Cu-$K_{\alpha 1}$ radiation (λ = 1.54506 Å) at 40.0 kV and 30.0 mA power. Measurements were performed over a 2θ range of 5–50°, using 0.7 mm glass capillaries. Capillaries were spun during measurements. The resulting diffractograms are reported in Figures 1 and 2. The crystallite size of both samples was calculated in two different ways: with the Scherrer formula[1,2] and *via* Rietveld refinement with TOPAS Academic (version *6*)[3], refining the parameter CS_G (crystallite size Gaussian). 5-ASA exhibits a calculated domain size between 51 nm (CS_G, Rietveld refinement) and 52 nm (Scherrer formula). S-NAP exhibits a calculated domain size between 49 nm (Scherrer formula) and 58 nm (CS_G, Rietveld refinement).

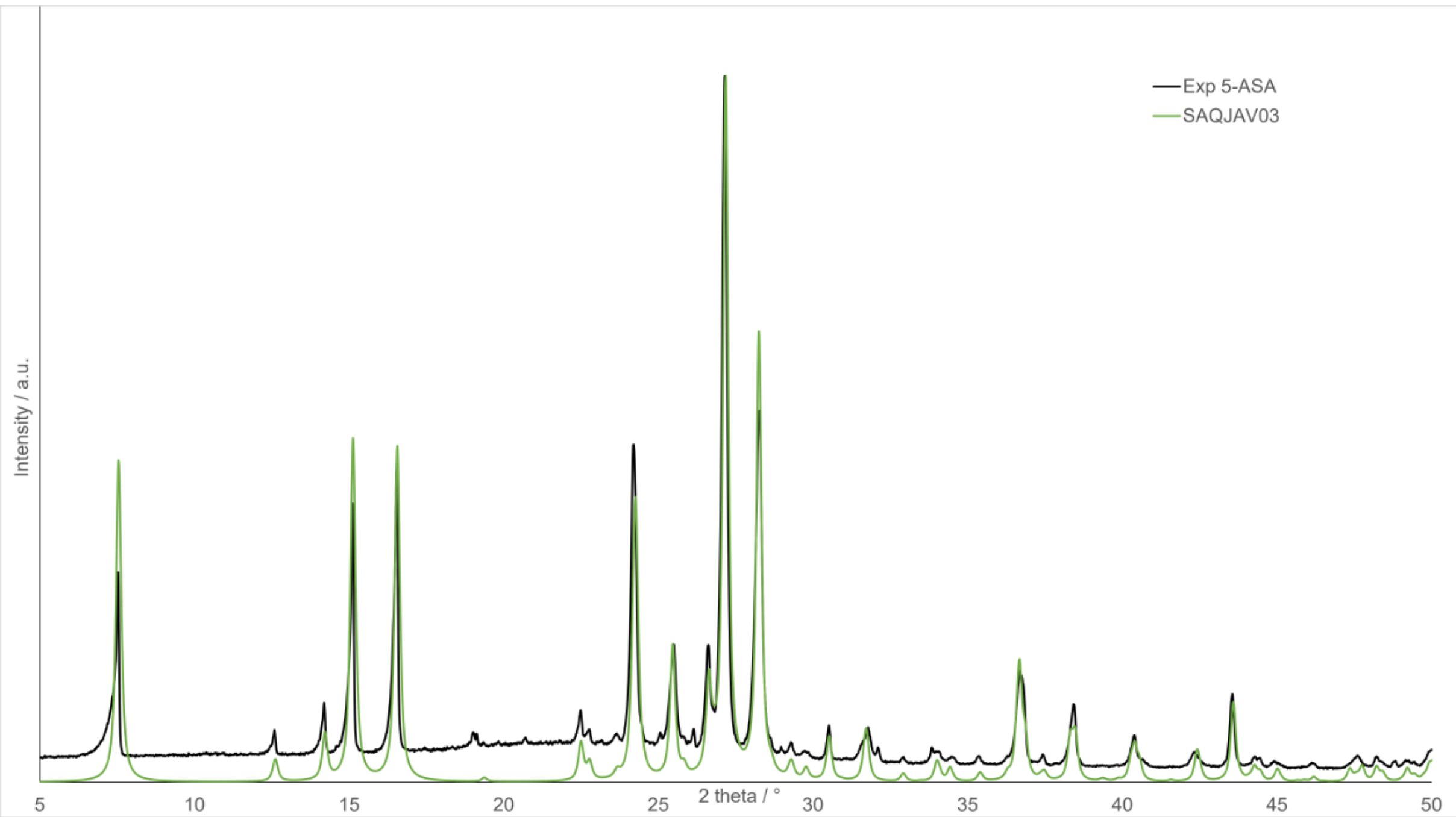

Figure 1. Powder patterns from experimental powder (black) and simulated from crystallographic data of the crystal structure SAQJAV03 (green) of 5-ASA in a 2θ range of 5–50°.

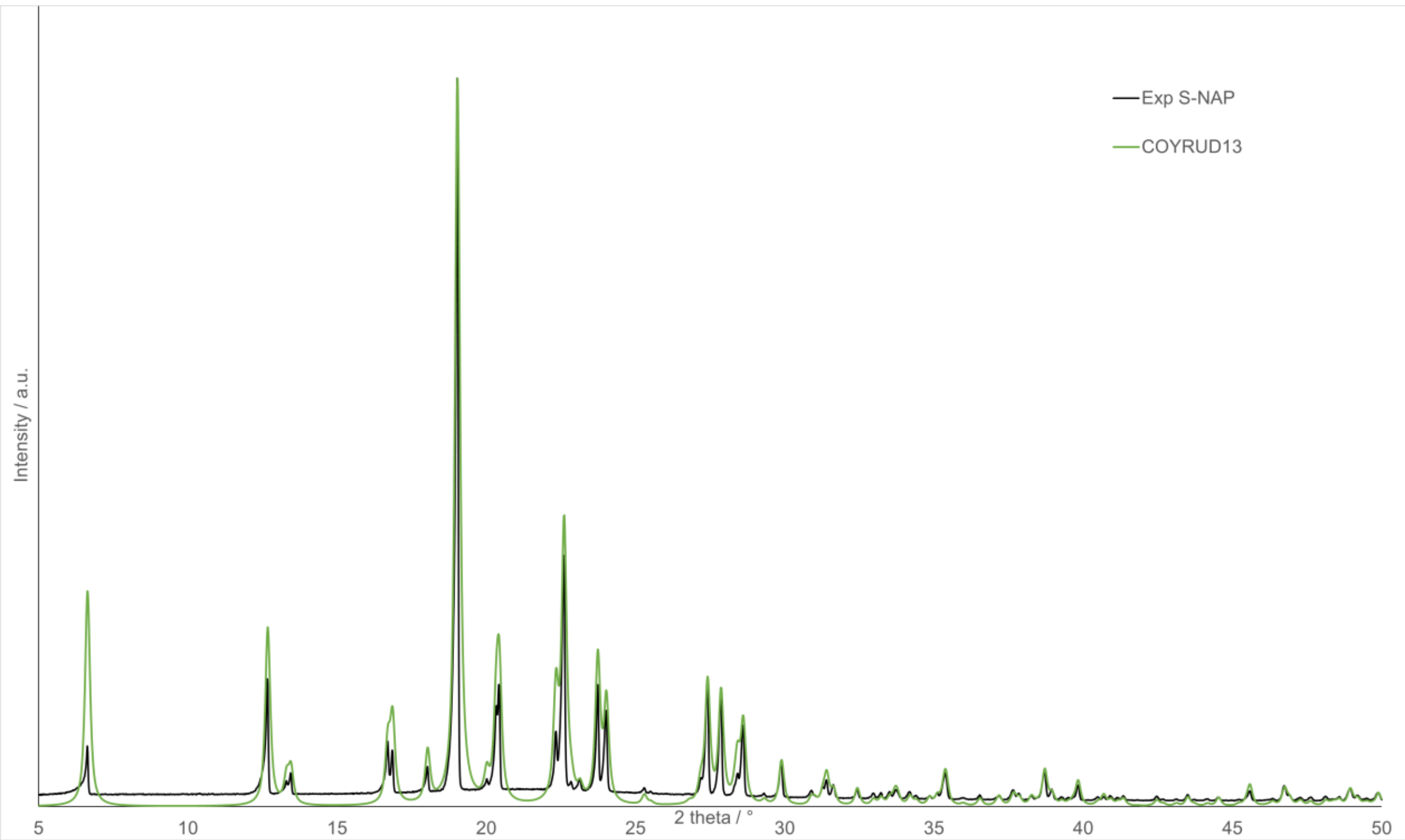

Figure 2. Powder patterns from experimental powder (black) and simulated from crystallographic data of the crystal structure COYRUD13 (green) of S-NAP in a 2θ range of 5–50°.

2.2 Synchrotron powder diffraction for Pair Distribution Function (PDF) refinement

Synchrotron measurements were performed at DESY, using the PETRA III P02.1 beamline.[4] The data were collected over a 2θ range of 0.5–63.5°, with a Varex XRD 4343CT detector using a wavelength of 0.207377 Å and a counting rate of 60 s per frame. For sample and background measurements, 1 mm glass capillaries were used. The acquired diffractograms are reported in Figures 3 and 4.

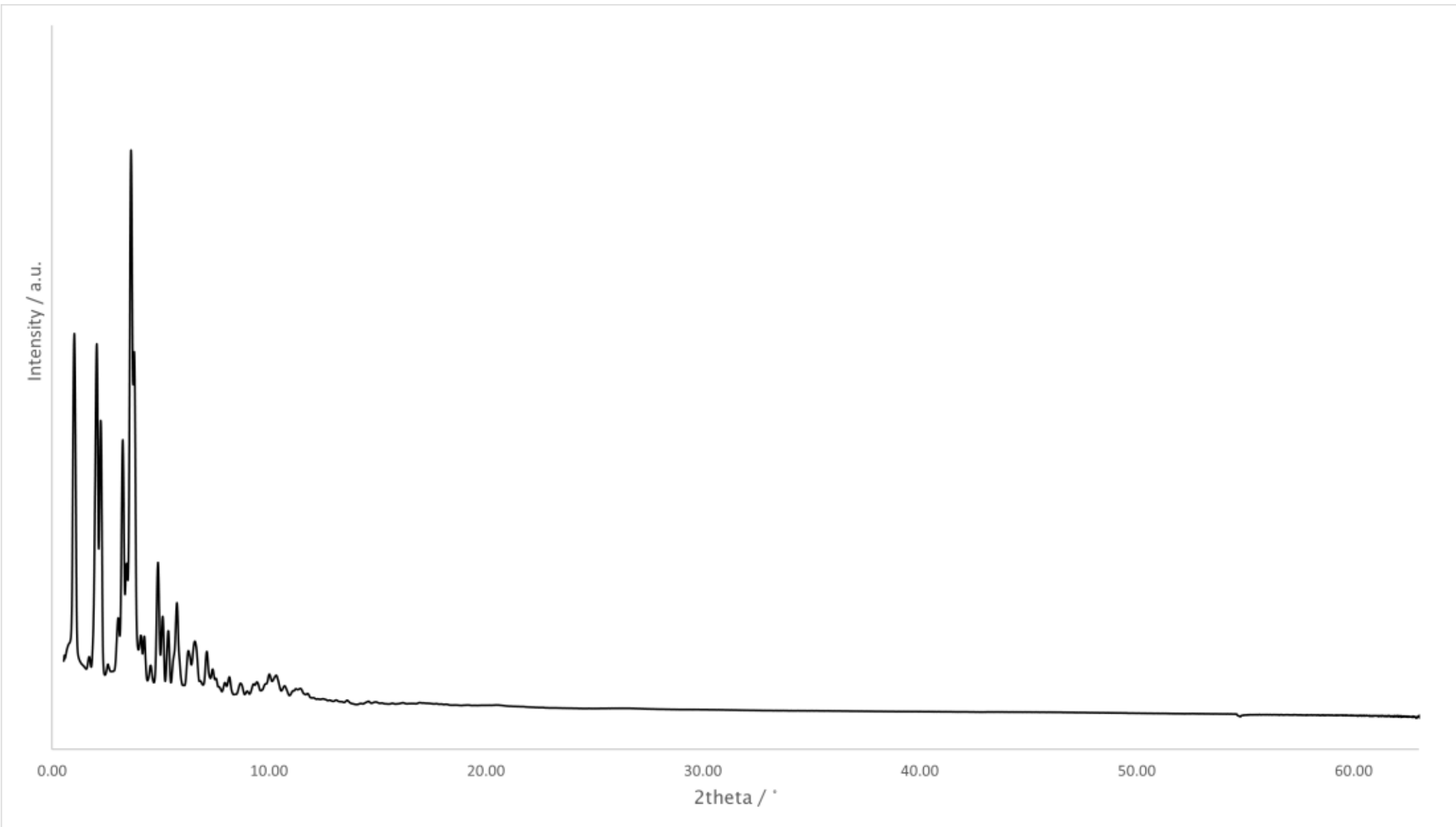

Figure 3. Synchrotron patterns of 5-ASA in a 2θ range of 0.5–63.5°.

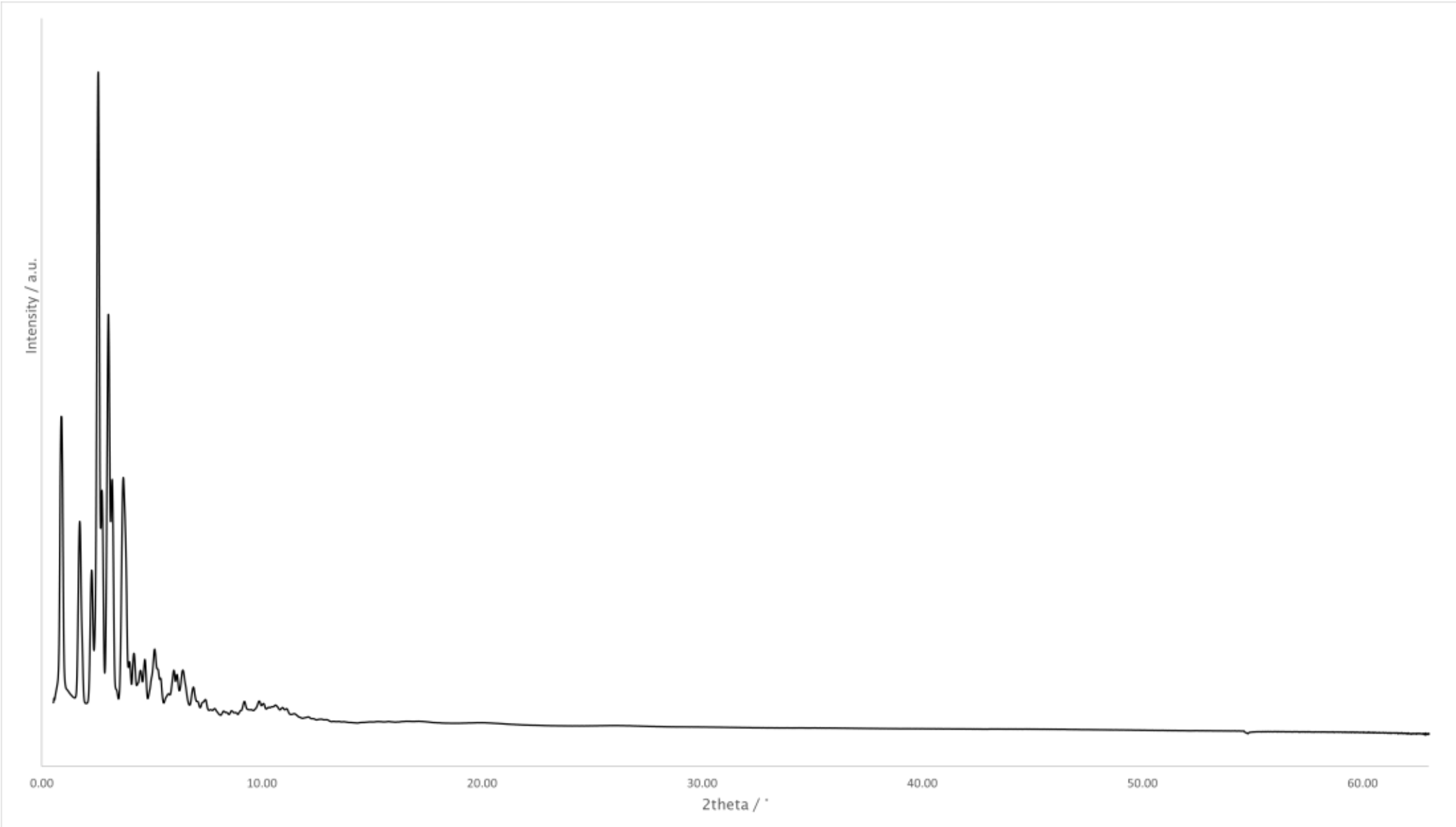

Figure 4. Synchrotron patterns of S-NAP in a 2θ range of 0.5–63.5°.

2.3 Obtaining the Pair Distribution Function (PDF)

The PDFs G(r) of the two samples were obtained from the synchrotron powder data by background correction, normalization, and Fourier transformation using the program PDFgetX3.[5] To exclude artefacts and insufficient statistics in the high 2θ range, the data were truncated at a finite maximum value of the momentum transfer Q_{max} = 20.0 $Å^{-1}$. The obtained PDFs are shown in Figures 5 and 6.

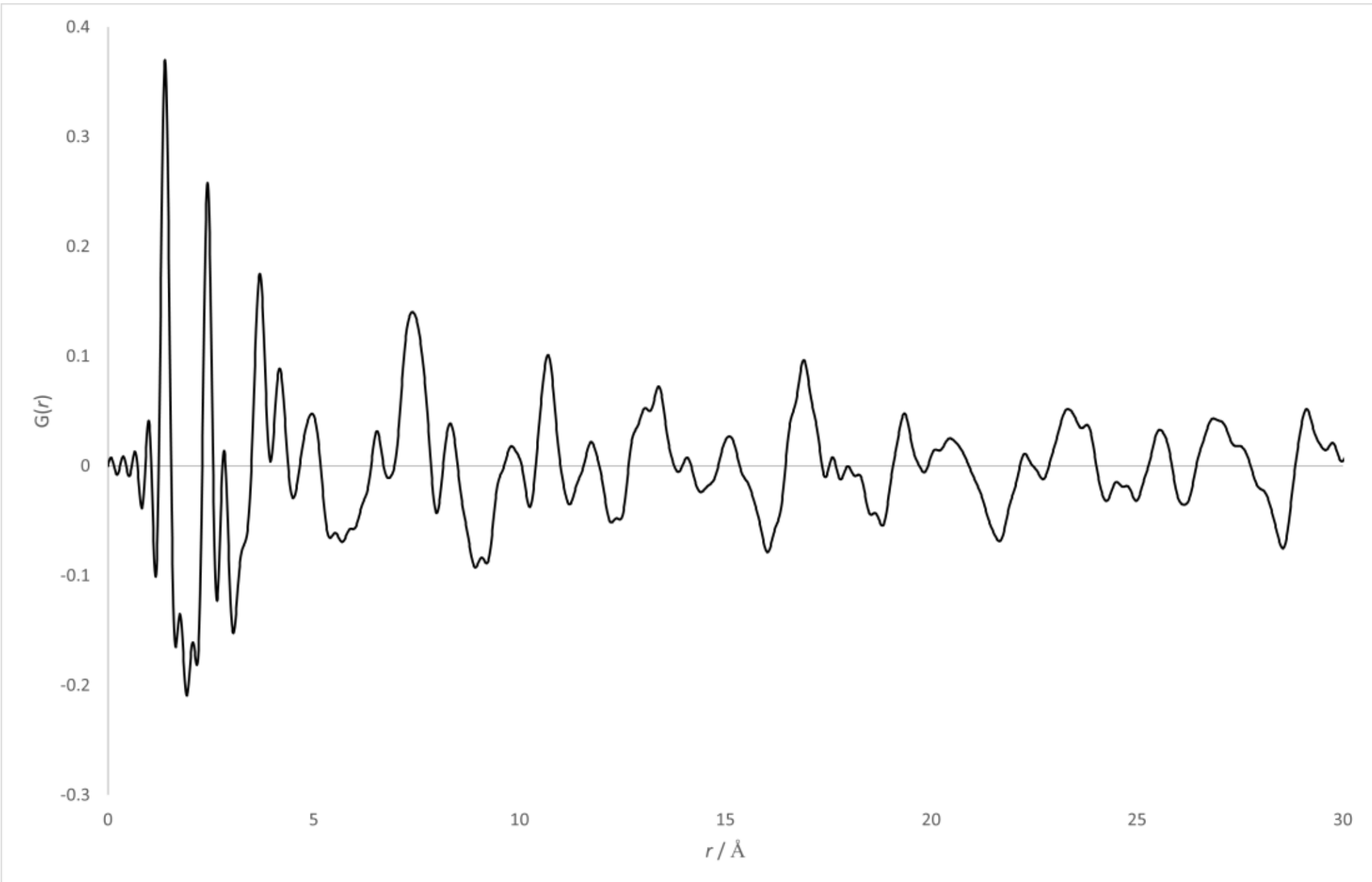

Figure 5. Experimental PDF curve of 5-ASA (Q_{max} = 20.0 $Å^{-1}$).

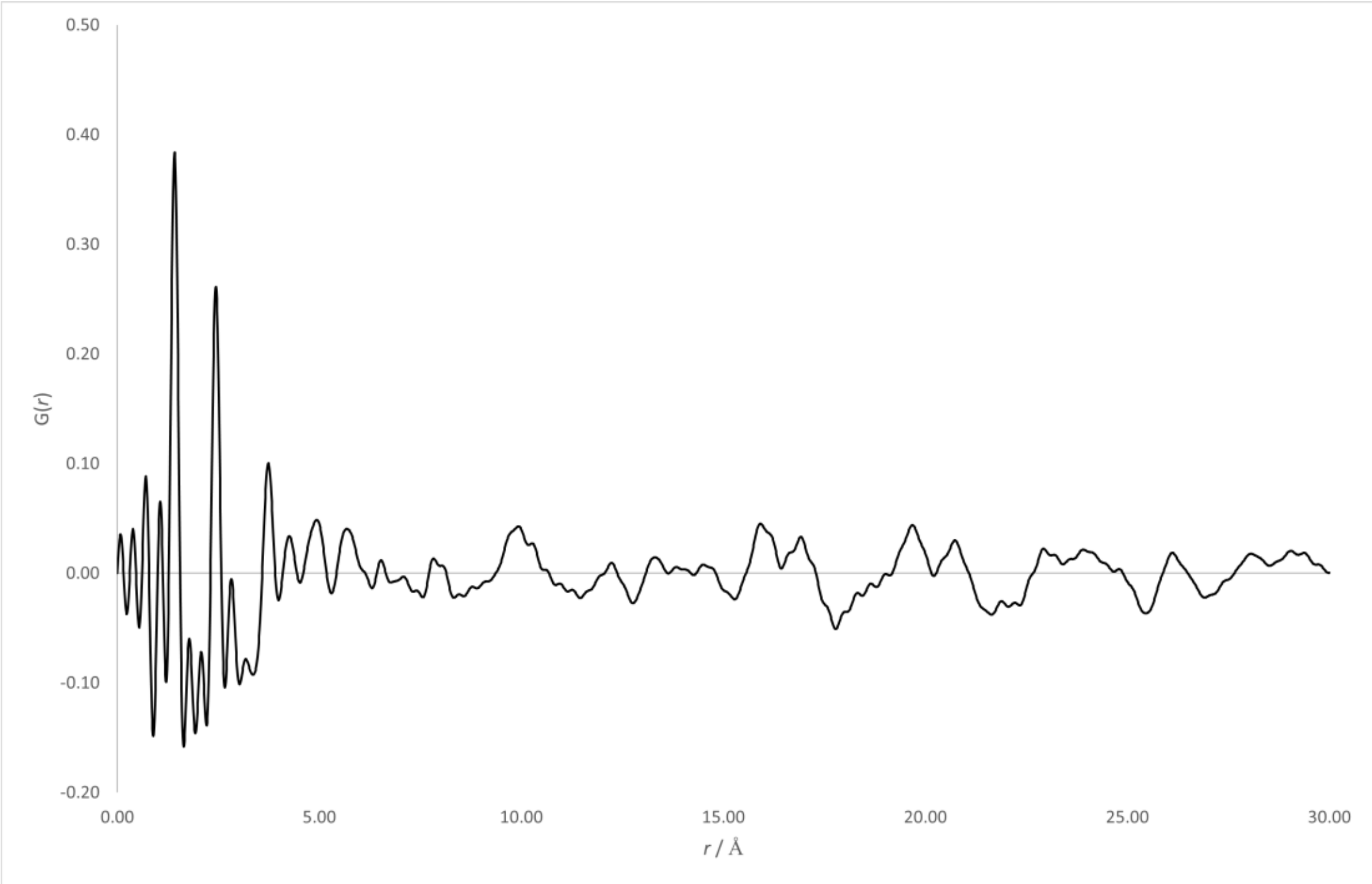

Figure 6. Experimental PDF curve of S-NAP (Q_{max} = 20.0 Å^{-1}).

The reproducibility of the PDF measurements was assessed by collecting independent measurements for the same sample using different capillaries. The overlays of the resulting PDFs for 5-ASA and S-NAP are shown in Figures 7 and 8, respectively.

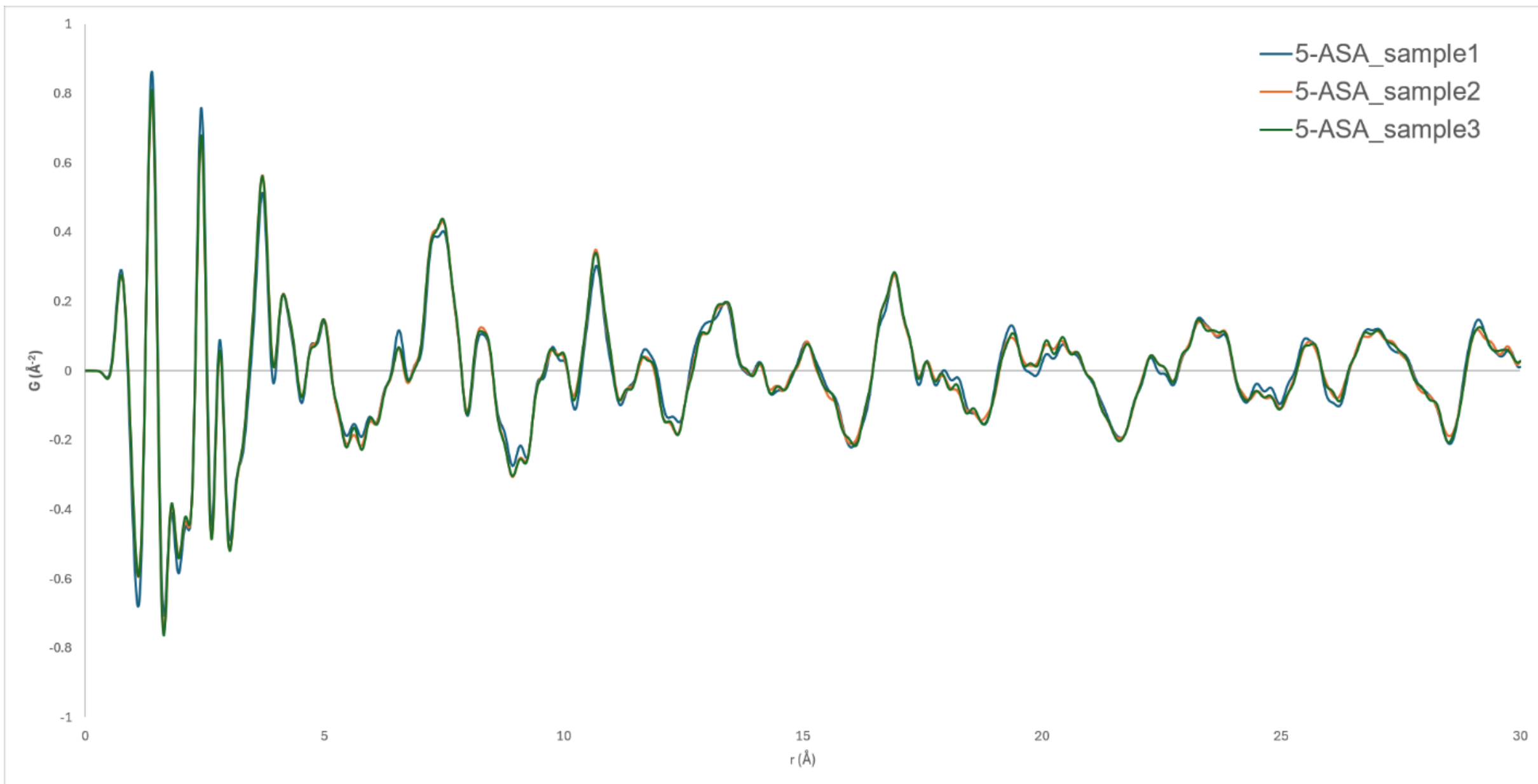

Figure 7. Overlay of the PDFs of three different measurements of the same sample of 5-ASA (Q_{max} = 20.0 Å^{-1}).

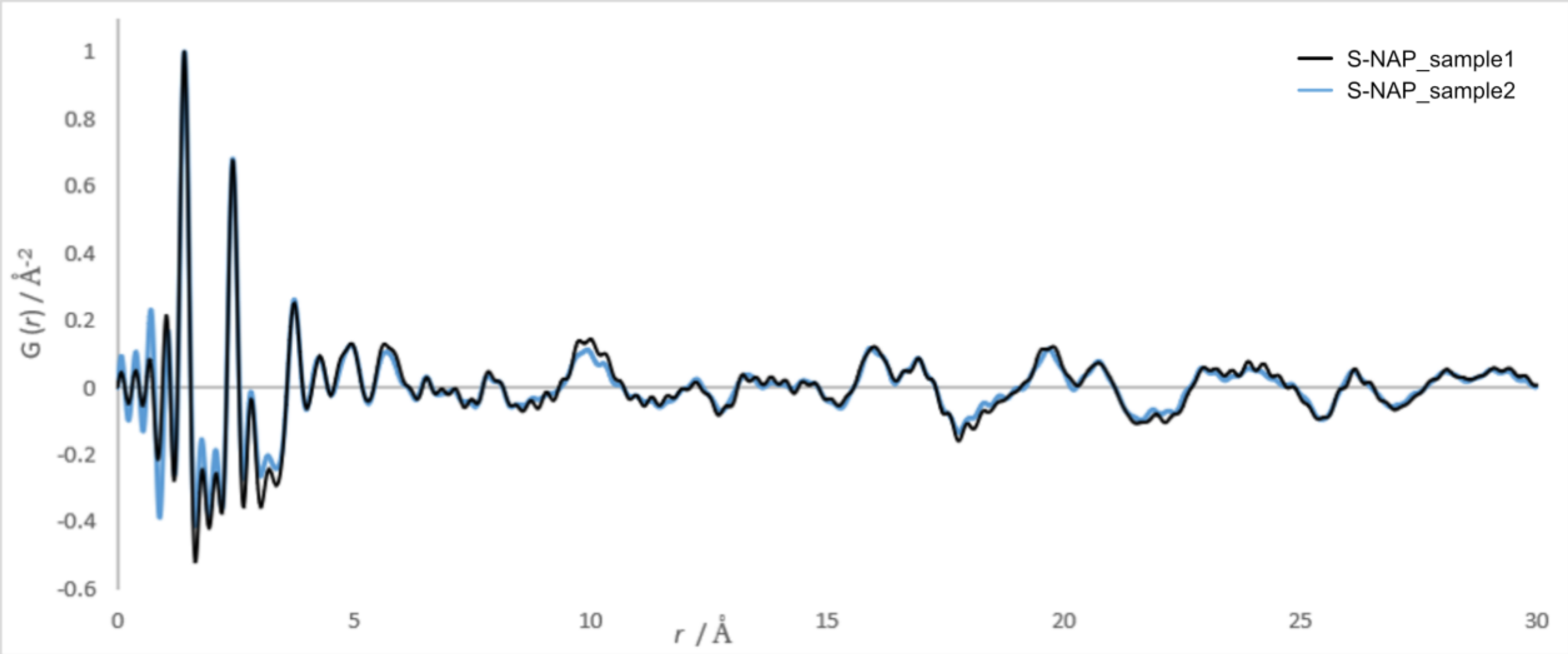

Figure 8. Overlay of the PDFs of two different measurements of the same sample of S-NAP (Q_{max} = 20.0 $Å^{-1}$).

The presence of some ripples in the PDF, in this case, does not affect the quality of the PDF fit. In any case, the number of ripples in the PDF was reduced as much as possible, while maintaining a sufficiently high Q_{max} to ensure high resolution.

3. Solid-state NMR (SSNMR)

All SSNMR spectra were collected at room temperature on a Jeol ECZR 600 instrument, operating at 600.17, 150.91, and 60.82 MHz for ^{1}H, ^{13}C, and ^{15}N nuclei, respectively.

^{13}C and ^{15}N CPMAS spectra were acquired using 3.2 mm zirconia rotors at a spinning speed of 20 and 9 kHz, respectively. A ramped cross-polarization pulse sequence was used with a 90° ^{1}H pulse of 2.0 and 2.5 μs, and a contact time of 3.5 and 4.0 ms for ^{13}C and ^{15}N, respectively. A two-pulse phase modulation (TPPM) decoupling scheme was used for ^{13}C, with a radiofrequency field of 125 kHz, while for ^{15}N a SPINAL64 decoupling scheme with a radiofrequency field of 100 kHz was applied. The ^{13}C and ^{15}N chemical shift scales were calibrated through the methylenic signal of the external standard adamantane ($\delta(^{13}C)$ = 38.48 ppm with reference to TMS) and the NH_3^+ signal of external standard α-glycine ($\delta(^{15}N)$ = 33.4 ppm with reference to liquid NH_3), respectively.

The ^{1}H MAS, ^{1}H-^{13}C Double-CP MAS, ^{1}H-^{14}N T-HMQC, and ^{1}H DQ/^{1}H SQ MAS spectra were acquired using 1 mm zirconia rotors at room temperature and at a spinning speed of 70 kHz. The ^{1}H radio frequency (rf) field strength was 285.7 kHz at a $\pi/2$ pulse length of 0.875 μs. The ^{1}H MAS spectra were acquired using a DEPTH[6] pulse sequence to suppress probe background and with optimized recycle delays (5*T_1 ^{1}H) of 6.0 and 30 s to ensure a quantitative response. The $^1H \rightarrow ^{13}C \rightarrow ^1H$ double cross polarization (^{1}H-^{13}C Double-CP) MAS experiments were performed with first contact time (ct1) of 2 ms, and second contact time (ct2) of 0.2 ms and 2 ms to investigate short- and long-range correlations, respectively. 14.3 kHz CW and 17.5 kHz WALTZ decouplings were applied during the t_1 and t_2 evolution time on the ^{1}H and ^{13}C channels, respectively. Phase alternated CW irradiation on the ^{1}H channel with 28.6 kHz radiofrequency field is applied during τ_z = 25 ms to suppress residual ^{1}H magnetization. The ^{1}H-^{14}N T-HMQC spectrum of 5-ASA was acquired using an optimized ^{14}N pulse length (τ_{mix}) of 0.4 ms. The ^{1}H DQ/^{1}H SQ MAS experiments were performed with the back-to-back (BABA) recoupling pulse sequence with excitation time durations of 16 rotor periods and with an optimized pulse length of 0.5 μs. The ^{1}H chemical shift scale was calibrated *via* the methylenic signal of the external standard alanine ($\delta(^1H)$ = 1.5 ppm with reference to TMS).

All recycle delays used in this work were optimized by means of saturation recovery experiments. Other SSNMR experimental parameters are reported in Table 1.

Table 1. ^{1}H MAS, ^{13}C and ^{15}N CPMAS, ^{1}H-^{13}C short- and long-range Double-CP MAS, ^{1}H-^{14}N T-HMQC, and ^{1}H DQ/^{1}H SQ MAS acquisition parameters for 5-ASA and S-NAP.

Experiment	Parameters	5-ASA	S-NAP
^{13}C CPMAS	acquisition time (ms)<br>n° scans<br>relaxation delay (s)<br>resolution (Hz)	29.49<br>1000<br>1<br>34	29.49<br>1440<br>6<br>34
^{15}N CPMAS	acquisition time (ms)<br>n° scans<br>relaxation delay (s)<br>resolution (Hz)	23.07<br>89780<br>1<br>43	-
^{1}H MAS	acquisition time (ms)<br>n° scans<br>relaxation delay (s)<br>resolution (Hz)	16.38<br>32<br>6<br>61	26.21<br>64<br>30<br>61
^{1}H-^{13}C Double-CP short range	acquisition time (ms)<br>n° increments<br>n° scans<br>relaxation delay (s)<br>^{1}H resolution (Hz)<br>^{13}C resolution (Hz)	16.38<br>128<br>48<br>1.6<br>61.0<br>294.8	16.38<br>128<br>24<br>6<br>61.0<br>294.8
^{1}H-^{13}C Double-CP long-range	acquisition time (ms)<br>n° increments<br>n° scans<br>relaxation delay (s)<br>^{1}H resolution (Hz)<br>^{13}C resolution (Hz)	16.38<br>128<br>96<br>1.6<br>61.0<br>294.8	16.38<br>128<br>48<br>6<br>61.0<br>294.8
^{1}H-^{14}N T-HMQC	acquisition time (ms)<br>n° increments<br>n° scans<br>relaxation delay (s)<br>^{1}H resolution (Hz)<br>^{14}N resolution (Hz)	8.19<br>64<br>48<br>1.6<br>122.1<br>1094.4	-
^{1}H DQ/^{1}H SQ MAS	acquisition time (ms)<br>n° baba<br>n° increments<br>n° scans<br>offset (ppm)<br>relaxation delay (s)<br>SQ resolution<br>DQ resolution	8.19<br>1<br>128<br>12<br>7.0<br>1.6<br>122.1<br>136.7	8.19<br>2<br>128<br>12<br>5.5<br>6<br>122.1<br>136.7

3.1 5-aminosalicylic acid (5-ASA)

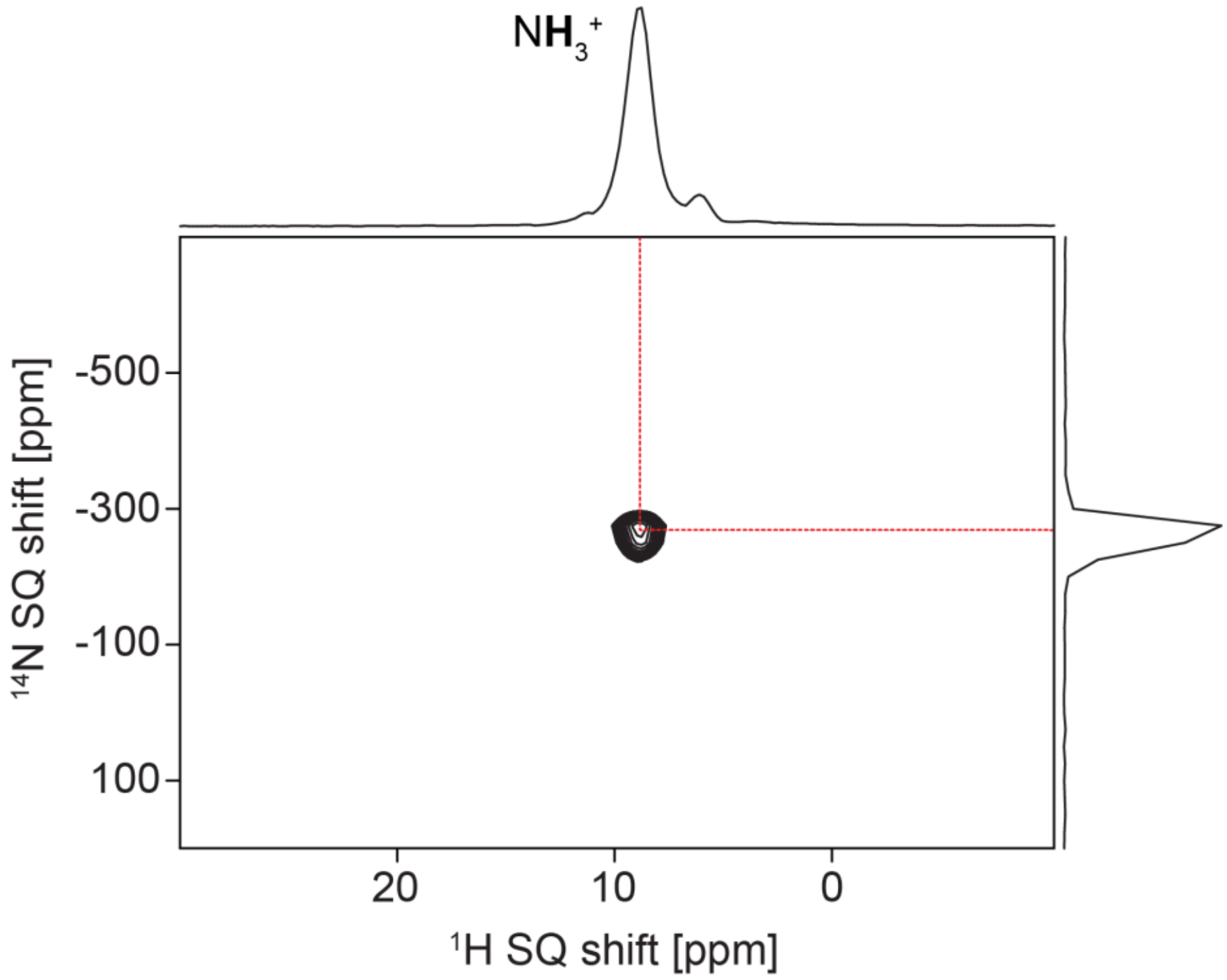

Figure 9. ^{1}H-^{14}N T-HMQC spectrum of 5-ASA acquired at room temperature at a spinning speed of 70 kHz. The correlation enabling the assignment of the protons of the protonated amine moiety is highlighted with dashed red lines.

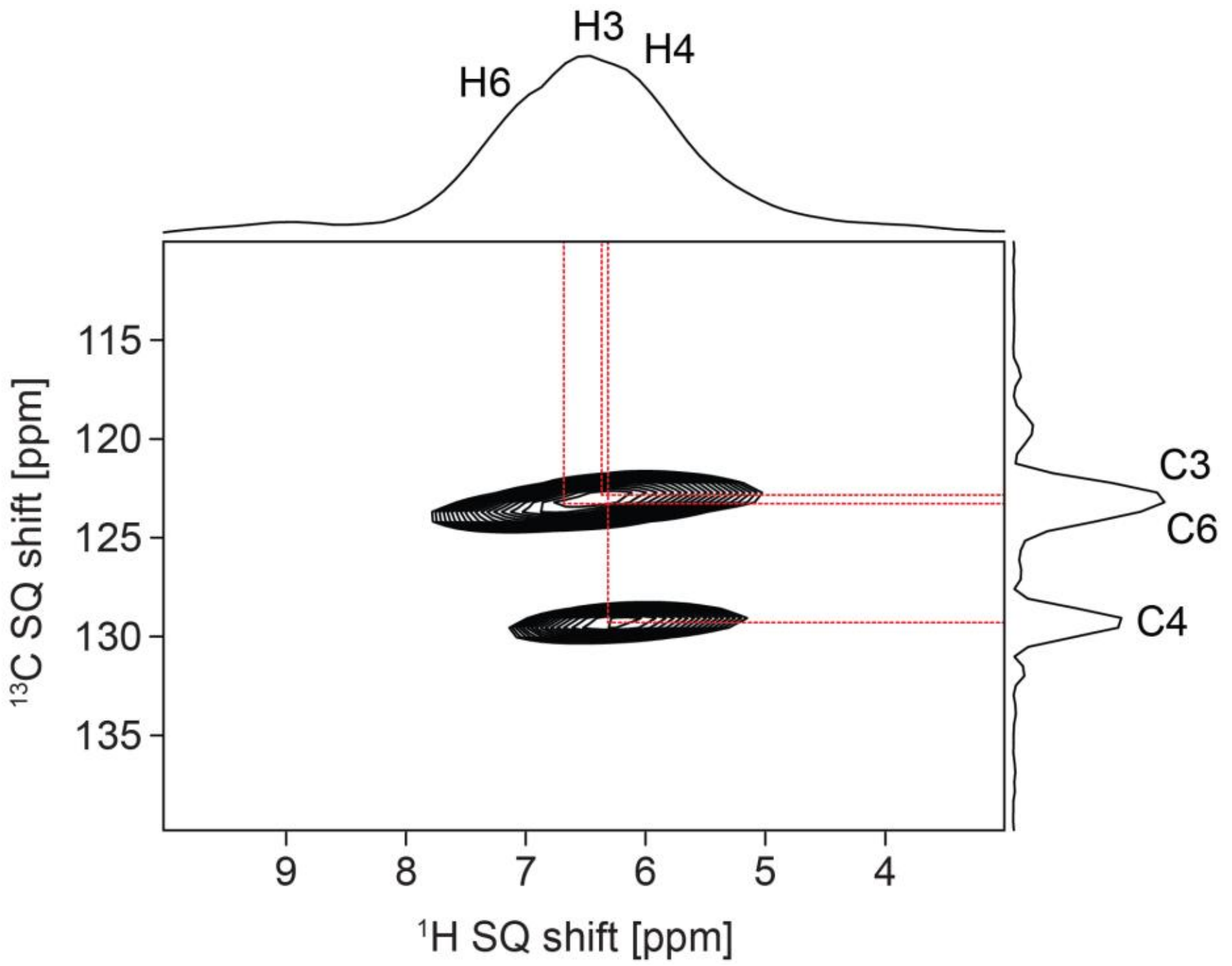

Figure 10. Short-range ^{1}H-^{13}C Double-CP spectrum of 5-ASA acquired at room temperature at a spinning speed of 70 kHz. The correlations between the CH carbons and their covalently bound protons are highlighted with dashed red lines.

Table 2. Experimental and computed ^{1}H, ^{13}C, and ^{15}N chemical shifts and corresponding assignment for 5-ASA.

^{1}H			^{13}C			^{15}N		
**Signal**	**δ exp (ppm)**	**δ calc (ppm)**	**Signal**	**δ exp (ppm)**	**δ calc (ppm)**	**Signal**	**δ exp (ppm)**	**δ calc (ppm)**
**OH**	11.4	12.1	C7	175.5	174.6	NH_3^+	44.6	46.4
NH_3^+	9.0	9.0	C2	161.8	162.6			
H6	6.7	6.9	C4	129.4	130.5			
H3	6.3	6.4	C6	123.8	123.8			
H4	6.3	6.5	C3	122.8	123.5			
			C5	119.5	119.6			
			C1	116.5	114.7			

Table 3. General assignment and corresponding integrals of the ^{1}H signals of the quantitative ^{1}H MAS spectrum of 5-ASA.

**δ (ppm)**	**Signal**	**Integral**
11.4	**OH**	1
9.0	NH_3^+	3
~6.6	Aromatic **H**	3

3.2 S-naproxen (S-NAP)

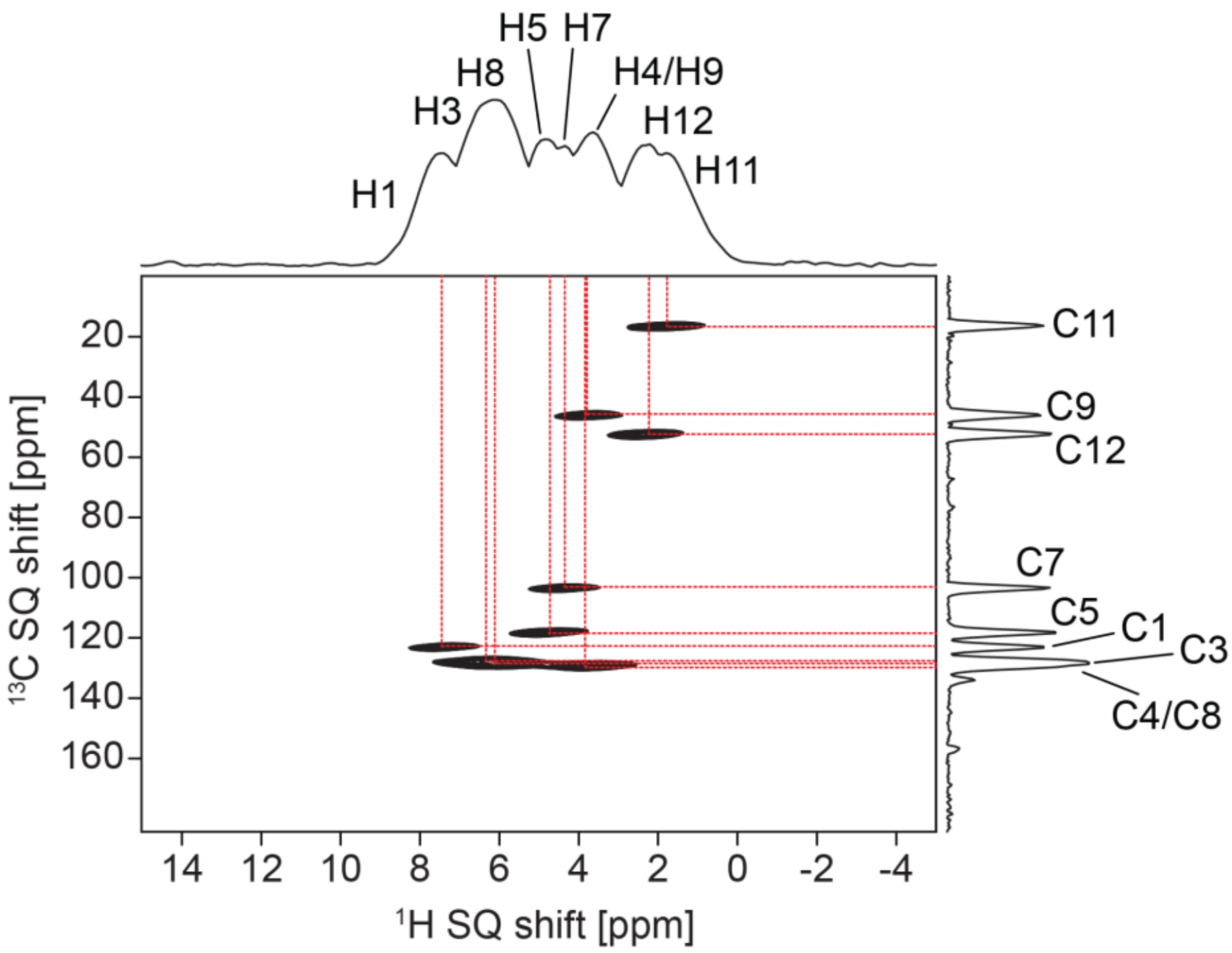

Figure 11. Short-range ^{1}H-^{13}C Double-CP spectrum of S-NAP acquired at room temperature at a spinning speed of 70 kHz. The correlations between the CH carbons and their covalently bound protons are highlighted with dashed red lines.

Table 4. Experimental and computed ^{1}H and ^{13}C chemical shifts and corresponding assignment for S-NAP.

^{1}H			^{13}C		
**Signal**	**δ exp (ppm)**	**δ calc (ppm)**	**Signal**	**δ exp (ppm)**	**δ calc (ppm)**
**OH**	11.5	12.4	C10	178.6	180.2
H1	7.4	7.0	C6	157.6	159.0
H3	6.3	5.8	C2	134.4	134.8
H8	6.0	5.2	C14	134.4	133.1
H5	4.8	4.5	C4	129.8	129.1
H7	4.3	4.1	C8	129.2	128.9
H4	3.7	3.6	C13	128.4	128.0
H9	3.7	3.4	C3	128.4	127.9
H12	2.2	2.8	C1	123.5	122.9
H11	1.7	1.6	C5	118.7	118.4
			C7	104.0	102.6
			C12	52.8	53.0
			C9	46.4	49.2
			C11	16.8	16.0

4. Motif search in Cambridge Structural Database (CSD)

4.1 5-aminosalicylic acid (5-ASA)

The motif search for the $S(6)$ motif was carried out using ConQuest (CSD version 5.45)[7], employing the following queries:

- Target structure:

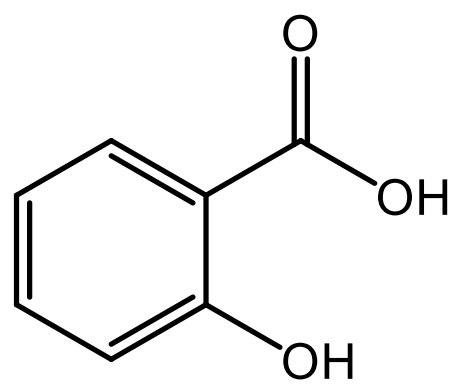

- Elements included: C, H, O, with other elements allowed
- Disorder and errors excluded
- Only structures determined by neutron diffraction

From the search results, 5 structures (out of 10 total hits) were identified after excluding the identical ones. These structures are summarized in Table 5, along with the corresponding hydrogen bond distances between donor and acceptor groups and bond angles within the $S(6)$ motif.

Table 5. Results of the motif search performed with ConQuest (CSD version 5.45).

Structure identifier	OH···O hydrogen bond distance* (Å)	OH···H hydrogen bond angle (°)
BESKAL11	2.573(2)	147.3(6)
GOKQUV07	2.551(5)	148.9(3)
GOKREG05	2.552(8)	150.4(0)
SALIAC03	2.603(1)	146.7(4)
ZZZEEU07	2.627(3)	145.4(5)

*The hydrogen bond distance refers to the distance between the heavy atoms involved in the hydrogen bond interaction.

4.2 S-naproxen (S-NAP)

The search for the analysis of H–H intermolecular distances in COOH···HOOC homodimers was carried out using ConQuest (CSD version 6.00)[7], employing the following queries:

- Analysed distances:

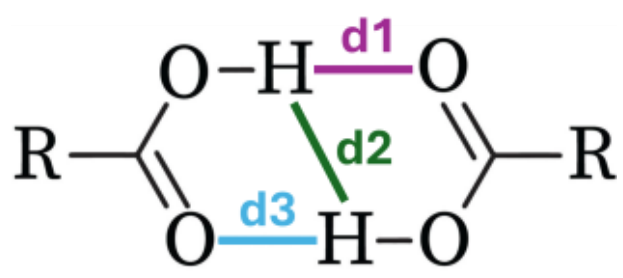

- Elements included: other elements allowed
- Disorder and errors excluded
- Only structures determined by neutron diffraction

The search resulted in 31 crystal structures. The results for the intermolecular H–H distance (d2) are reported in Figure 10. The calculated average distance is ~2.3 Å.

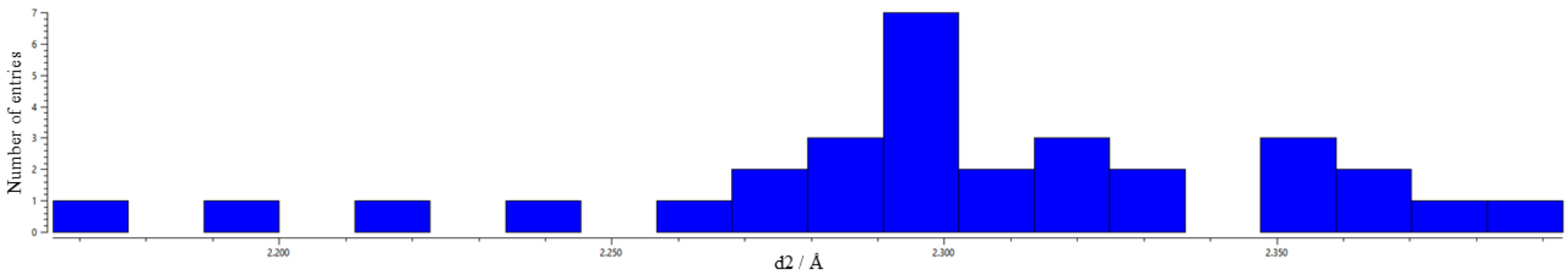

Figure 12. Histogram plot of the intermolecular H–H distance for the 31 entries of the ConQuest search.

5. FIDEL parameters

5.1 Lattice parameter ranges for random structure generation

5.1.1 5-aminosalicylic acid (5-ASA)

Table 6. Lattice parameter ranges used during structure generation of 5-ASA.

Space group	Z	Lattice parameters
$P2_1/c$	4	a min = 3 Å<br>a max = 19.7 Å<br><br>b min = 3 Å<br>b max = 39.4 Å<br><br>c min = 3 Å<br>c max = 39.4 Å<br><br>β min = 90°<br>β max = 120°
$P1$	1	a min = 3 Å<br>a max = 9.9 Å<br><br>b min = 3 Å<br>b max = 9.9 Å<br><br>c min = 3 Å<br>c max = 9.9 Å<br><br>α min = 90°<br>α max = 120°<br><br>β min = 90°<br>β max = 120°<br><br>γ min = 90°<br>γ max = 120°

$P\bar{1}$	2	a min = 3 Å<br>a max = 19.7 Å<br><br>b min = 3 Å<br>b max = 19.7 Å<br><br>c min = 3 Å<br>c max = 19.7 Å<br><br>α min = 90°<br>α max = 120°<br><br>β min = 90°<br>β max = 120°<br><br>γ min = 90°<br>γ max = 120°
$P2_1$	2	a min = 3 Å<br>a max = 19.7 Å<br><br>b min = 3 Å<br>b max = 19.7 Å<br><br>c min = 3 Å<br>c max = 19.7 Å<br><br>β min = 90°<br>β max = 120°
$P2_12_12_1$	4	a min = 3 Å<br>a max = 39.4 Å<br><br>b min = 3 Å<br>b max = 39.4 Å<br><br>c min = 3 Å<br>c max = 39.4 Å

5.1.2 S-naproxen (S-NAP)

Table 7. Lattice parameter ranges used during structure generation of S-NAP.

Space group	Z	Lattice parameters
$P2_1$	2	a min = 3 Å<br>a max = 29.6 Å<br><br>b min = 3 Å<br>b max = 29.6 Å<br><br>c min = 3 Å<br>c max = 29.6 Å<br><br>β min = 90°<br>β max = 120°
$P1$	1	a min = 3 Å<br>a max = 14.8 Å<br><br>b min = 3 Å<br>b max = 14.8 Å<br><br>c min = 3 Å<br>c max = 14.8 Å<br><br>α min = 90°<br>α max = 120°<br><br>β min = 90°<br>β max = 120°<br><br>γ min = 60°<br>γ max = 120°
$P2_12_12_1$	4	a min = 3 Å<br>a max = 59.1 Å<br><br>b min = 3 Å<br>b max = 59.1 Å<br><br>c min = 3 Å<br>c max = 59.1 Å

5.2 Comparison of experimental and simulated PDFs using cross-correlation functions

After removing the structures exhibiting atomic overlap, the remaining hundreds of thousands of structures (Table 8) were compared to the experimental PDFs using the similarity measure S_{12} in an elaborated automated multi-step process, which includes ranking according to the similarity to PDF using cross-correlation functions, clustering and removal of duplicates. A detailed description of the comparison of PDFs using cross-correlation functions is given by Habermehl *et al.*.[8–10]

S_{12} can take values from 0 to 1, where the value of 1 corresponds to identical PDFs. Even if a S_{12} threshold of 1 could theoretically be used, the main aim of the PDFs comparison step is to rule out unreasonable

structural models, rather than selecting the correct one. For this reason, S_{12} thresholds are usually set to values close to 1, *e.g.*, 0.98, which allows for the selection of promising structural models, which will be then fitted to the PDF, while maintaining reasonable computational time. Different S_{12} thresholds can be used in different runs, based on the computational resources and time available. The S_{12} thresholds used for the PDFs comparison of 5-ASA and S-NAP are reported in Table 9.

Table 8. Number of structures generated and compared to the experimental PDF using a cross-correlation function for 5-ASA and S-NAP. The total number of generated structures, before checking for atomic overlap, is even higher (~10^6). Plots of the comparison of the simulated and experimental PDFs for all the structures are available on request.

**5-ASA**		**S-NAP**	
**Run**	**Number of structures**	**Run**	**Number of structures**
R1c	11258	R1a	9999
R1d	2849	R1b	1571
R1e	2685	R1c	1433
R1f	6665	R1d	2873
R1g	4140	R1e	72803
R1h	9893	R1f	9999
R1i	84999	R1g	12659
R1j	14999	R1h	15981
R1k	19999	R2a	817
R1m	9422	R2b	2202
R2a	6383	R2c	2487
R2b	6252	R2d	2135
R2c	7038	R2e	2177
R2d	59999	R2f	2174
R3a	13484	R3a	3814
R3b	13603	R3b	3782
R3c	7452	R3c	4018
R4a	2815	R3d	25943
R4b	2557		
R4c	3291		
R4d	14999		
R5a	2298		
R5b	2807		
R5c	2965		
Total number of generated structures	312852	Total number of generated structures	176867

Table 9. FIDEL calculation parameters for structure generation of 5-ASA and S-NAP.

5-ASA			S-NAP		
**Run**	**space group**	S_{12} threshold	**Run**	**space group**	S_{12} threshold
R1c	$P2_1/c$	0.97	R1a	$P2_1$	0.98
R1d	$P2_1/c$	0.97	R1b	$P2_1$	0.965
R1e	$P2_1/c$	0.97	R1c	$P2_1$	0.98
R1f	$P2_1/c$	0.975	R1d	$P2_1$	0.98
R1g	$P2_1/c$	0.975	R1e	$P2_1$	0.985
R1h	$P2_1/c$	0.975	R1f	$P2_1$	0.98
R1i	$P2_1/c$	0.975	R1g	$P2_1$	0.98
R1j	$P2_1/c$	0.975	R1h	$P2_1$	0.98
R1k	$P2_1/c$	0.975	R2a	$P2_12_12_1$	0.96
R1m	$P2_1/c$	0.975	R2b	$P2_12_12_1$	0.97
R2a	$P\bar{1}$	0.97	R2c	$P2_12_12_1$	0.97
R2b	$P\bar{1}$	0.97	R2d	$P2_12_12_1$	0.97
R2c	$P\bar{1}$	0.97	R2e	$P2_12_12_1$	0.97
R2d	$P\bar{1}$	0.985	R2f	$P2_12_12_1$	0.97
R3a	$P1$	0.97	R3a	$P1$	0.97
R3b	$P1$	0.97	R3b	$P1$	0.97
R3c	$P1$	0.97	R3c	$P1$	0.97
R4a	$P2_1$	0.97	R3d	$P1$	0.97
R4b	$P2_1$	0.97			
R4c	$P2_1$	0.97			
R4d	$P2_1$	0.98			
R5a	$P2_12_12_1$	0.97			
R5b	$P2_12_12_1$	0.97			
R5c	$P2_12_12_1$	0.97			

5.3 PDF fit using TOPAS

After the comparison of experimental and simulated PDFs, 2241 structure candidates for 5-ASA, and 2418 for S-NAP, were subjected to an automated multi-step fit to the PDF using TOPAS Academic (version *6*)[3]. All parameters used and refined in the PDF fit are reported in Table 10.

Table 10. FIDEL-TOPAS PDF-fit algorithm used for calculations of both 5-ASA and S-NAP. Legend: x = gets refined; n.a. = not applied; fixed = no refinement, value fixed to result from previous refinement; fixed to V = no refinement, value fixed to given value (system/sample dependent)*; macro pdf_mode 2 = "for simulated annealing with z-Matrix and B_{iso}-intra/B_{iso}-inter; macro pdf_mode 0 = "for refinement with Bintra/Binter (default)".

Parameters	Step 1	Step 2	Step 3	Step 4	Step 5	Step 6	Step 7	Step 8
macro_pdf_mode n.	n.a	2	2	2	2	2	2	2
number of iterations	100	2000	2500	2500	3000	3000	3000	3000
zero-point error	n.a	x	x	x	x	x	x	x
scale factor	n.a	x	x	x	x	x	x	x
molecule rotation	x	fixed	x	x	x	x	x	x
molecule translation	x	fixed	x	x	x	x	x	x
dampening function	n.a	fixed to V	fixed	fixed	x	x	x	x
B_{iso}-intra/B_{iso}-inter	n.a	fixed to V	fixed	fixed	x	x	x	x
lattice parameters	fixed	x	x	fixed	x	x	x	x
molecular DOF**	n.a	n.a	n.a	n.a	n.a	n.a	n.a	n.a

*Values for dampening and B-intra/B-inter are fixed to the values found in the local PDF-fit for the correct crystal structure. If the correct crystal structure is unavailable, the value from the average of previous FIDEL runs can be used.

**DOF: degrees of freedom

6. Application of SSNMR constraints for structure filtering

Table 11. Results of the structure filtering using the H–H (OH···OH and OH···NH_3^+) intermolecular contacts in the 0–4.00 Å range as constraints for 5-ASA. The label "Starting structures" refers to the structures that exhibited a good agreement after the comparison of the simulated and experimental PDFs using a cross-correlation function, and not to the total number of generated structures.

Run	Starting structures	Erased structures	Remaining structures
R1c	200	109	91
R1d	100	55	45
R1e	100	59	41
R1f	100	64	36
R1g	100	51	49
R1h	100	61	39
R1i	8	4	4
R1j	81	46	35
R1k	27	13	14
R1m	100	50	50
R2a	100	55	45
R2b	100	58	42
R2c	100	47	53
R3a	150	65	85
R3b	150	73	77
R3c	100	39	61
R4a	100	66	34
R4b	100	67	33
R4c	100	65	35
R4d	25	12	13
R5a	100	77	23
R5b	100	77	23
R5c	100	66	34
Total number of structures	2241	1279	962

Table 12. Results of the structure filtering using the H–H (OH···OH, OCH_3···H1, and OCH_3···OH) intermolecular contacts in the 0–4.00 Å range as constraints for S-NAP. The label "Starting structures" refers to the structures that exhibited a good agreement after the comparison of the simulated and experimental PDFs using a cross-correlation function, and not to the total number of generated structures.

Run	Starting structures	Erased structures	Remaining structures
R1a	80	75	5
R1b	100	97	3
R1c	100	98	2
R1d	150	146	4
R1e	149	143	6
R1f	92	89	3
R1g	149	143	6
R1h	148	145	3
R2a	100	90	10
R2b	150	132	18
R2c	150	140	10
R2d	150	137	13
R2e	150	142	8
R2f	150	136	14
R3a	150	149	1
R3b	150	144	6
R3c	150	148	2
R3d	148	144	4
Total number of structures	2418	2300	118

Table 13. Result of the structures filtering on the best 40 structures of 5-ASA, ranked according to the R_{wp}^{PDF}. Structures highlighted in red were discarded according to SSNMR constraints.

Run	Number	Space Group	R_{wp}^{PDF}
R1k	5348	$P2_1/c$	33.09
R3a	3269	P1	36.35
R3a	4770	P1	37.48
R3a	9385	P1	37.74
R3a	11814	P1	38.07
R3a	8396	P1	38.26
R3a	4273	P1	38.30
R1i	48369	$P2_1/c$	38.38
R3a	12130	P1	38.46
R3a	9624	P1	38.83
R3a	336	P1	39.20
R3a	12919	P1	39.24
R3a	11149	P1	39.28
R1f	6112	$P2_1/c$	39.47
R3a	9334	P1	39.54
R3a	6400	P1	39.78
R3a	12408	P1	39.79
R1e	2151	$P2_1/c$	39.89
R1f	630	$P2_1/c$	39.89
R3a	8751	P1	40.02
R2b	2464	P-1	40.14
R1e	1654	$P2_1/c$	40.25
R3a	4523	P1	40.34
R1f	1837	P21/c	40.36
R3a	7016	P1	40.38
R3a	82	P1	40.48
R3a	4937	P1	40.58
R3a	2010	P1	40.60
R3a	3497	P1	40.76
R2c	1132	P-1	40.77
R1f	5437	$P2_1/c$	40.78
R3a	1417	P1	40.86
R1e	1415	$P2_1/c$	40.90
R3a	2230	P1	40.93
R3a	8906	P1	40.95
R3a	5584	P1	40.97
R3a	12012*	P1	41.04
R1e	288	$P2_1/c$	41.09
R3a	583	P1	41.13
R1f	3118	$P2_1/c$	41.13

*Identical to structure R3a_4937

Table 14. Result of the structures filtering on the best 40 structures of S-NAP, ranked according to the R_{wp}^{PDF}. Structures highlighted in red were discarded according to SSNMR constraints.

Run	Number	Space Group	R_{wp}^{PDF}
R1b	1041	$P2_1$	34.86034
R1h	8277	$P2_1$	34.95297
R2c	2166	$P2_12_12_1$	35.21699
R2c	366	$P2_12_12_1$	36.75927
R2d	512	$P2_12_12_1$	37.06619
R2a	27	$P2_12_12_1$	37.47162
R2d	141	$P2_12_12_1$	37.49738
R2c	2021	$P2_12_12_1$	38.26451
R2a	358	$P2_12_12_1$	38.41074
R2b	140	$P2_12_12_1$	38.84542
R2a	365	$P2_12_12_1$	38.88636
R2c	405	$P2_12_12_1$	39.11938
R2d	526	$P2_12_12_1$	39.15459
R2d	1644	$P2_12_12_1$	39.22696
R2a	12	$P2_12_12_1$	39.45393
R2a	717	$P2_12_12_1$	39.89675
R2c	556	$P2_12_12_1$	40.11916
R2c	2007	$P2_12_12_1$	40.14761
R2a	382	$P2_12_12_1$	40.34281
R2a	618	$P2_12_12_1$	40.43153
R2a	474	$P2_12_12_1$	40.56207
R2b	561	$P2_12_12_1$	40.72655
R2a	58	$P2_12_12_1$	40.78421
R2a	504	$P2_12_12_1$	40.88546
R2d	1841	$P2_12_12_1$	40.9061
R2a	413	$P2_12_12_1$	40.93039
R2a	379	$P2_12_12_1$	40.9473
R2b	988	$P2_12_12_1$	40.96123
R2d	416	$P2_12_12_1$	41.16406
R2c	783	$P2_12_12_1$	41.17293
R2d	1163	$P2_12_12_1$	41.18915
R2c	1040	$P2_12_12_1$	41.24488
R2c	579	$P2_12_12_1$	41.39422
R1a	6220	$P2_1$	41.40587
R2c	76	$P2_12_12_1$	41.4102
R1d	2664	$P2_1$	41.43353
R2a	17	$P2_12_12_1$	41.49699
R1g	2473	$P2_1$	41.54018
R2d	2067	$P2_12_12_1$	41.65237
R1f	687	$P2_1$	41.80136

7. DFT-D optimisation and SSNMR chemical shifts calculation

The crystal structures were optimised at DFT-D level with Quantum Espresso (QE, v. 6.4.1),[11] employing the projector augmented wave (PAW) approach, with the non-local vdW-df2 method[12] and the B86r functional[13] with the SSSP set of pseudopotentials[14]. An energy cut-off of 60 Ry was used. Starting from the optimised structures with fixed lattice parameters, NMR calculations were performed using the gauge including projected augmented wave (GIPAW)[15] and the PBE pseudopotentials from PS Library 1.0.0[16] with an energy cut-off of 80 Ry, following the methodology previously described.[17,18] The theoretical absolute isotropic magnetic shielding (σ_{iso}) values of the sample were converted into isotropic chemical shifts (δ_{iso}) by applying the linear regression method.[19,20]

To visualize and generate pictures of the experimental and optimised crystal structures, the Mercury[21] software was employed. To compare the optimised and experimental crystal structures, the Cartesian RMSD (root-mean square deviation) value was calculated, using the Crystal Packing Similarity tool available in the Mercury software. For the comparison, a molecular cluster of 20 molecules was used, applying a 20% tolerance on both distances and angles. Hydrogen atoms positions were not considered.

Table 15. Results of the DFT-D optimisation with fixed cell parameters for the best 10 structures of 5-ASA. The structures are ranked according to their relative lattice energy.

Structure	ΔE / kJ/mol	a / Å	b / Å	c / Å	α /°	β /°	γ /°	Vol / Å^3
SAQJAV03*	/	3.7688	7.3492	23.4893	90.0000	94.6555	90.0000	648.452
R1k_5348	0.00	3.7646	7.3520	23.5616	90.0000	95.3400	90.0000	649.292
R1f_6112	17.31	3.7446	7.3129	25.9121	90.0000	113.4179	90.0000	651.142
R3a_4273	41.36	3.7477	6.4377	7.5798	117.4320	90.9255	94.1327	161.666
R3a_8396	41.93	3.7450	6.4430	7.3300	66.0500	85.6100	85.5800	160.958
R3a_9624	51.90	3.7600	5.7620	7.4740	77.1700	78.3400	83.5600	154.240
R3a_9385	64.71	3.8340	6.3710	7.1200	76.9700	80.7100	88.0300	167.215
R1i_48369	70.76	6.4514	7.6571	14.4837	90.0000	114.1799	90.0000	652.671
R3a_9334	80.66	3.8120	6.5630	7.4100	97.0000	91.0900	106.0300	176.578
R3a_12919	101.60	3.8160	6.6060	6.4780	107.9000	90.7400	106.0600	148.472
R3a_12130	150.58	3.9530	6.6040	7.4170	117.8100	102.0800	107.6800	148.348

*Original cell setting is $P2_1/n$. The cell setting was transformed into $P2_1/c$ for better comparison.

Table 16. Results of the DFT-D optimisation with fixed cell parameters for the best 10 structures of S-NAP. The structures are ranked according to their relative lattice energy.

Structure	ΔE / kJ/mol	a / Å	b / Å	c / Å	α /°	β /°	γ /°	Vol / Å^3
COYRUD13	/	7.8759	5.7834	13.3230	90.0000	93.8770	90.0000	605.467
R1b_1041	0.00	7.8939	5.8062	13.3299	90.0000	93.7900	90.0000	609.612
R1h_8277*	0.02	7.9360	5.7890	13.3070	90.0000	93.8500	90.0000	609.964
R1d_2664**	5.86	7.8380	5.8130	13.4600	90.0000	94.1000	90.0000	1108.230
R1f_687	9.24	5.8210	7.8040	13.9940	90.0000	97.5500	90.0000	630.196
R2d_1647	25.41	5.6800	7.9050	28.3900	90.0000	90.0000	90.0000	1274.722
R2a_12	35.93	5.5714	7.9660	24.3934	90.0000	90.0000	90.0000	1082.390
R2a_382	44.46	27.8789	7.8423	5.8127	90.0000	90.0000	90.0000	1270.930
R2a_413	54.14	5.7551	12.0391	15.7969	90.0000	90.0000	90.0000	1094.490
R2c_405	57.55	7.8376	5.8132	13.4596	90.0000	90.0000	90.0000	611.699
R1g_2473	62.25	11.2160	5.7650	7.8740	90.0000	104.9700	90.0000	491.856

*R1h_8277 is identical to R1b_1041 post-DFT-D optimisation.

**R1d_2664 exhibits the same molecular arrangement of R1b_1041 post-DFT-D optimisation, with just small deviations of the lattice parameters and a slight molecular distortion.

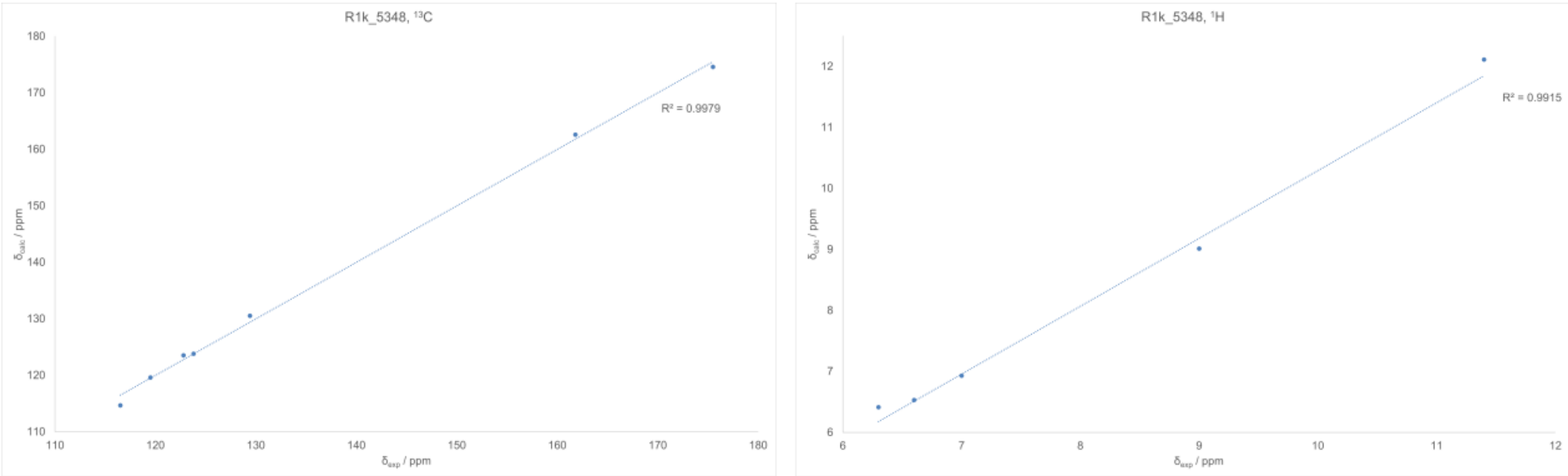

Figure 13. Plots of the calculated *vs* experimental ^{13}C and ^{1}H chemical shifts for the best candidate structure of 5-ASA, R1k_5348.

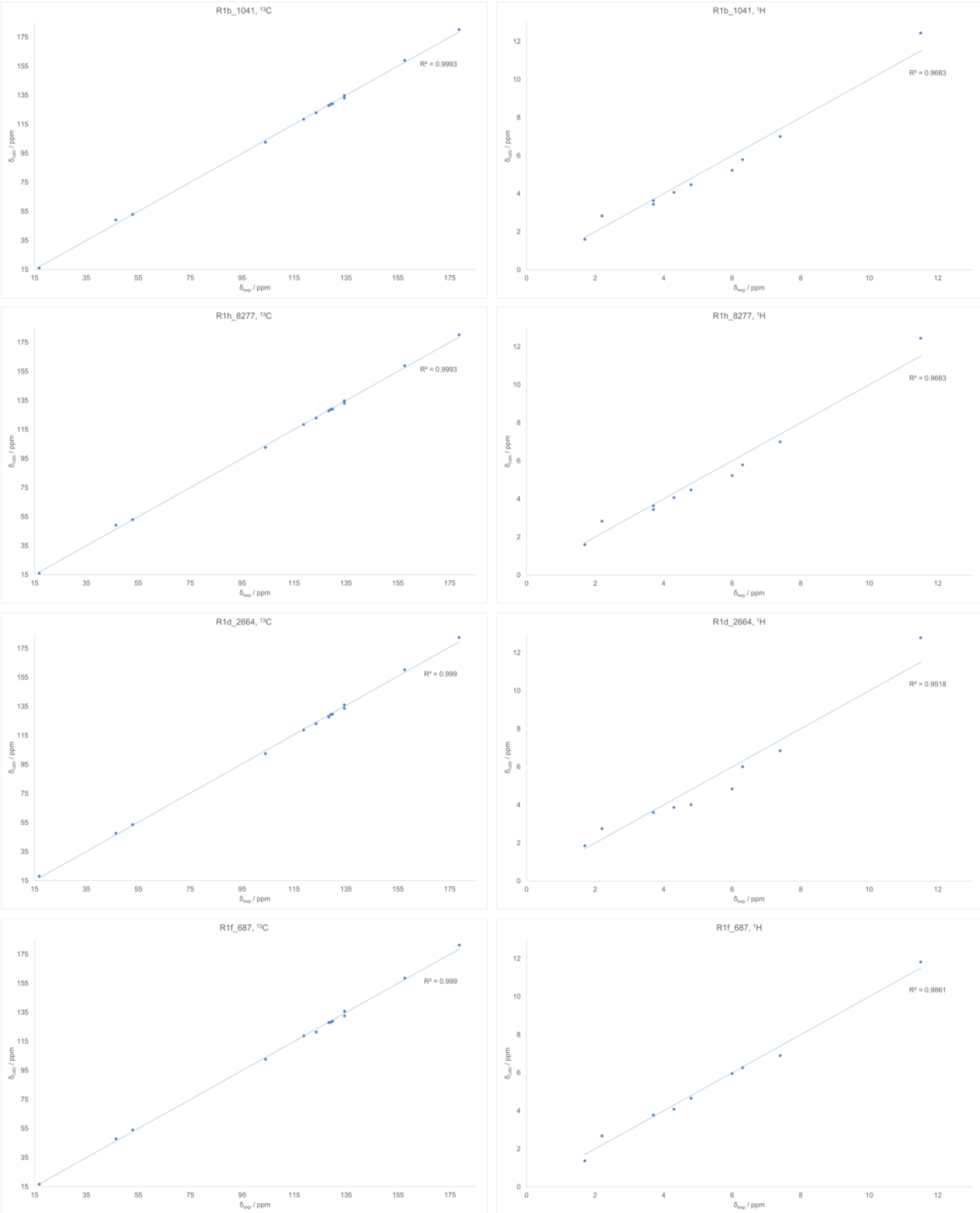

Figure 14. Plots of the calculated *vs* experimental ^{13}C and ^{1}H chemical shifts for the best four candidate structures of S-NAP.

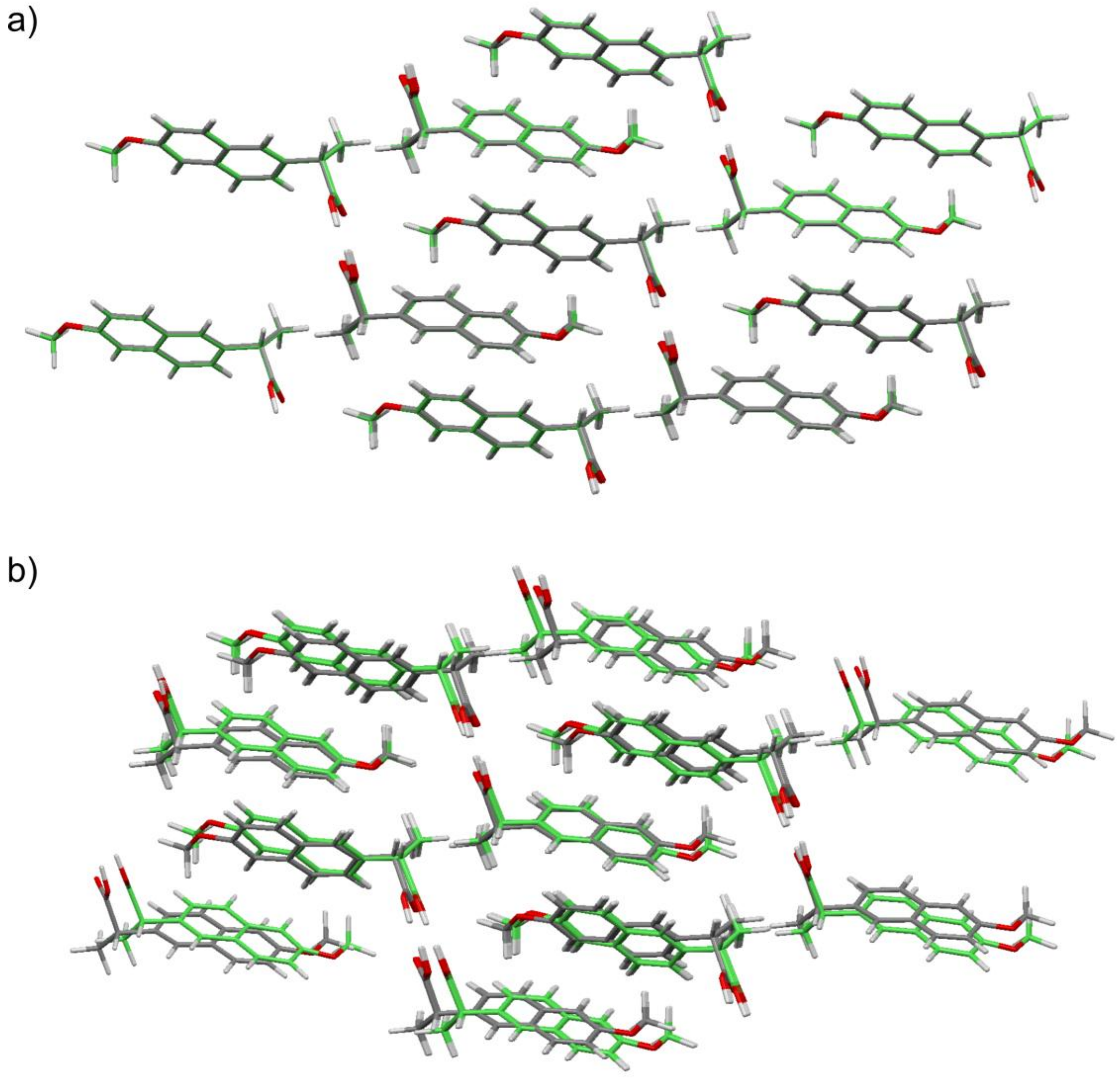

Figure 15. Overlay of structure candidate R1b_1041 (standard colors) with (a) structure candidate R1h_8277 (green) and (b) structure candidate R1d_2664 (green). The overlay was done with the Crystal Packing Similarity tool of Mercury, considering a cluster of 30 molecules and a tolerance of 20%. The resulting RMSCD values are 0.033 Å and 0.633 Å for R1h_8277 and R1d_2664, respectively.

8. PDF-fit

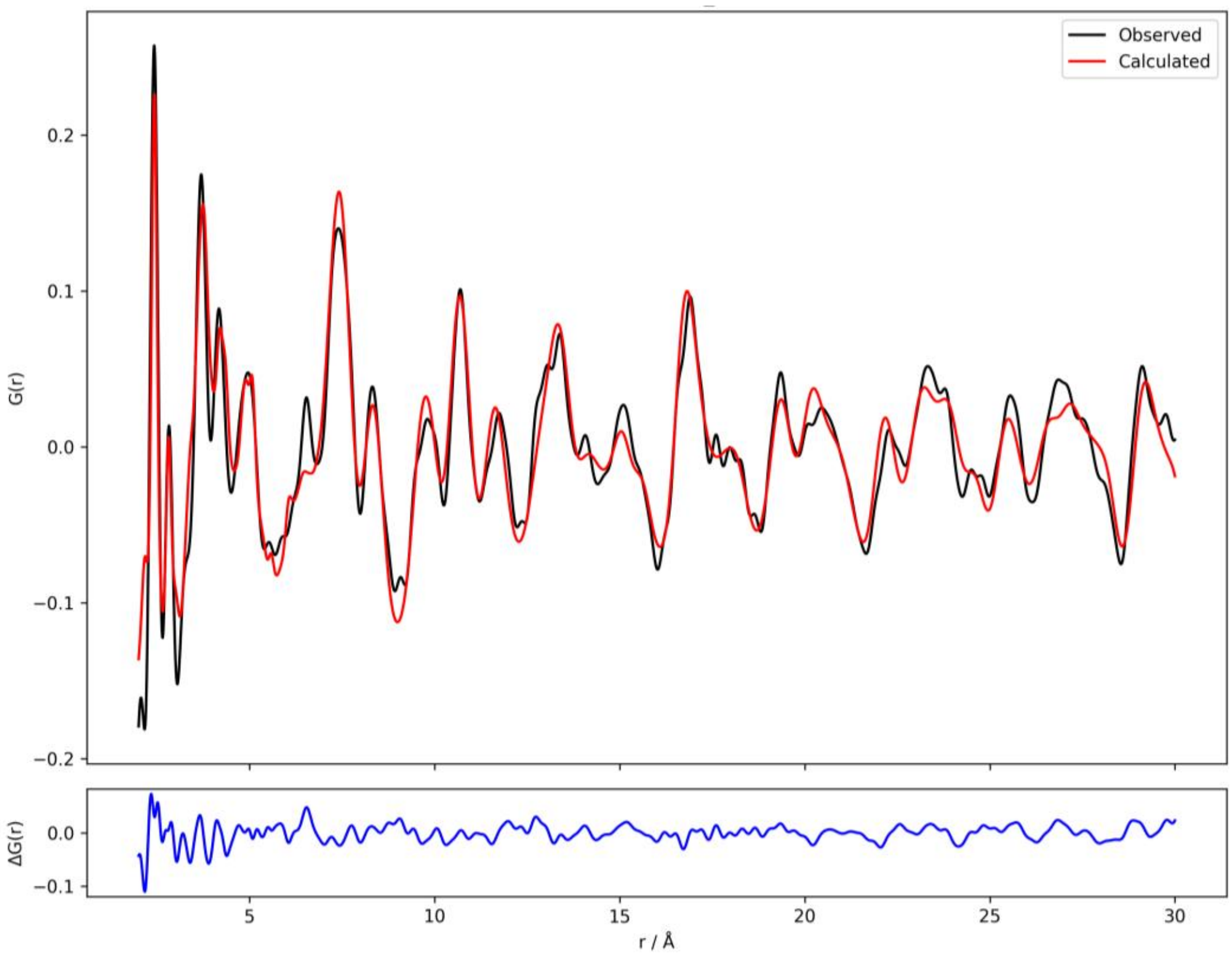

Figure 16. PDF-fit plot of structure R1k_5348 (5-ASA). Experimental PDF in black, calculated PDF in red, and difference curve in blue.

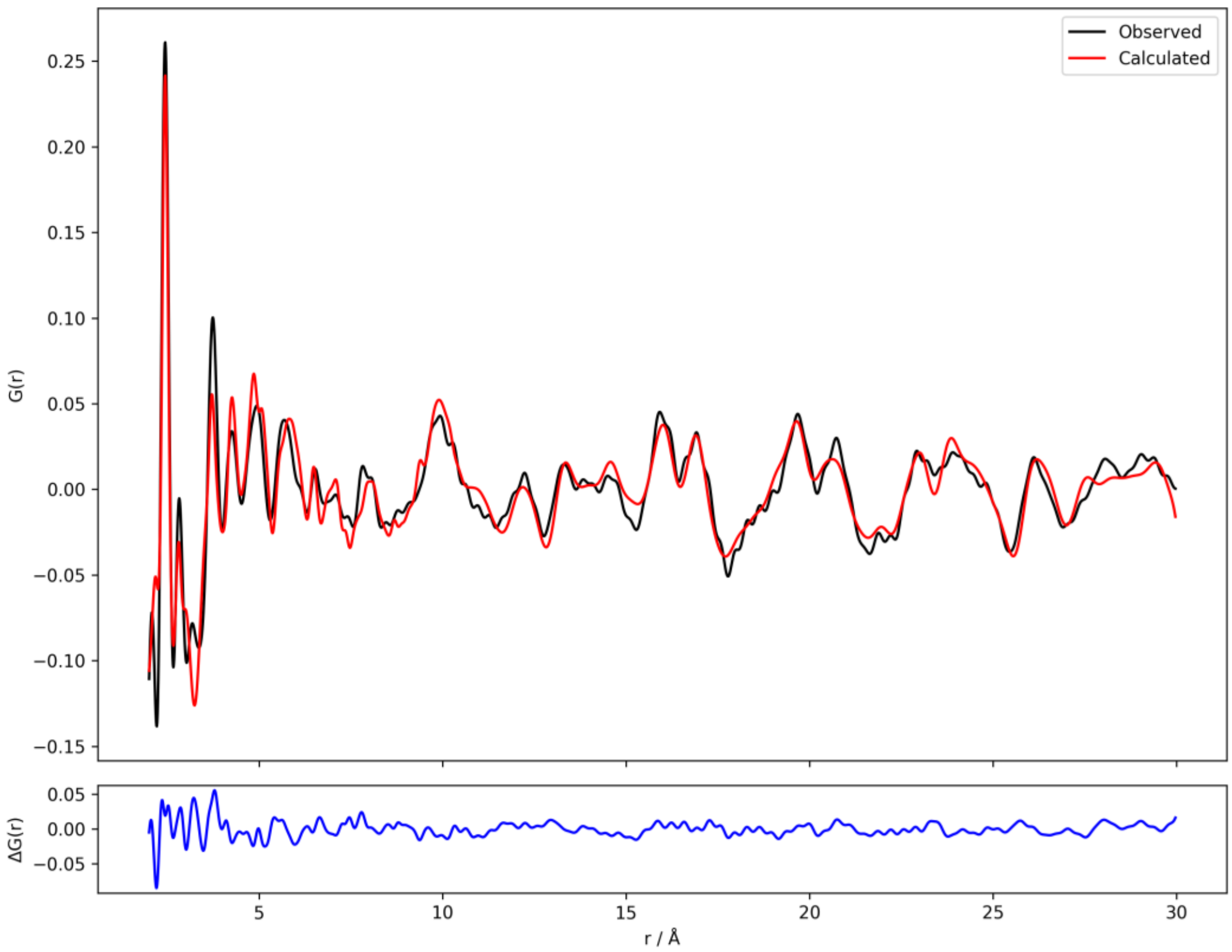

Figure 17. PDF-fit plot of structure R1b_1041 (S-NAP). Experimental PDF in black, calculated PDF in red, and difference curve in blue.

9. Comparison of experimental and simulated powder patterns for structure confirmation

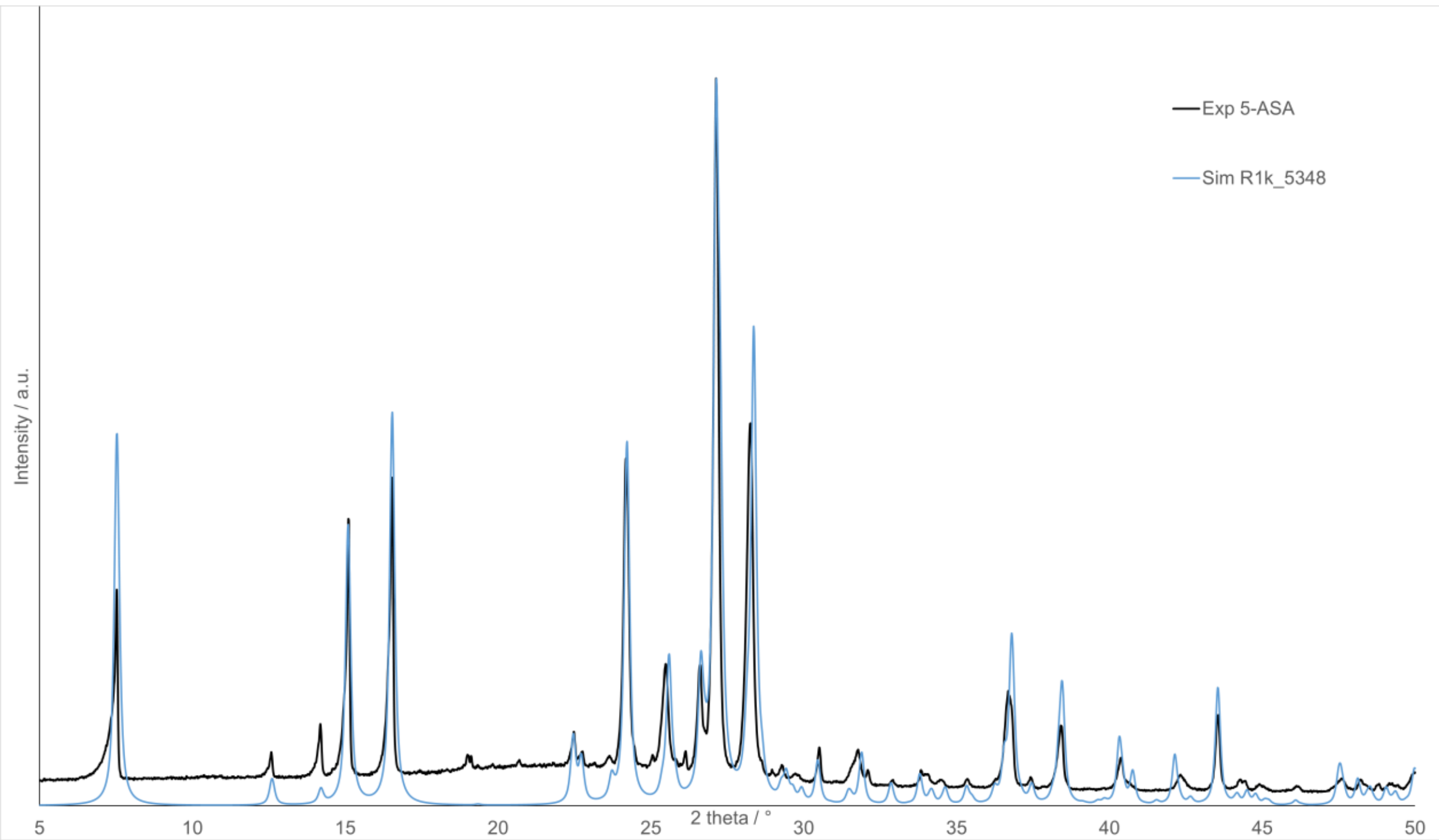

Figure 18. Comparison of the experimental powder pattern of 5-ASA (Exp 5-ASA) with the simulated powder pattern of the candidate structure R1k_5348 (Sim R1k_5348).

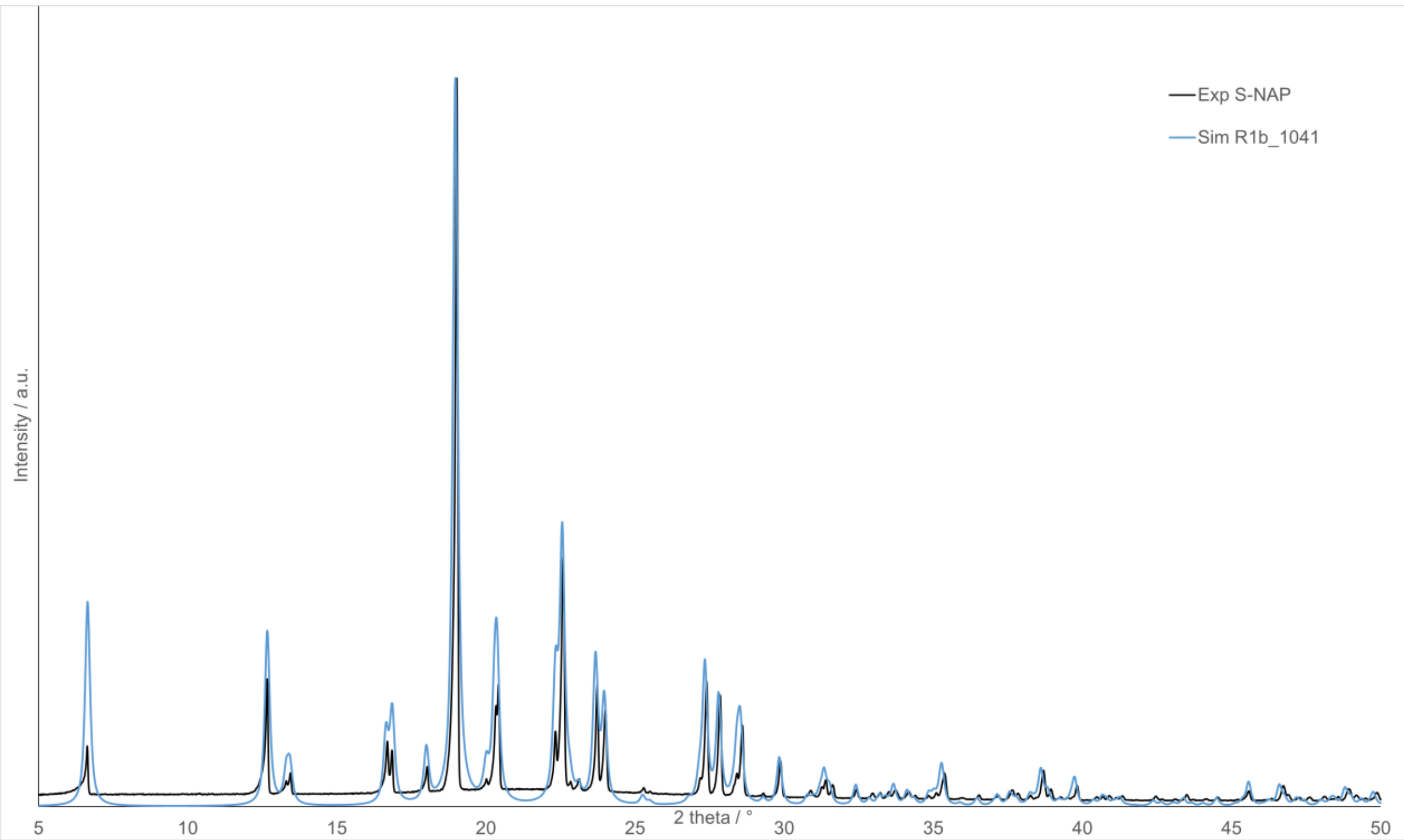

Figure 19. Comparison of the experimental powder pattern of S-NAP (Exp S-NAP) with the simulated powder pattern of the candidate structure R1b_1041 (Sim R1b_1041).

References

1. Scherrer, P. Bestimmung der Größe und der inneren Struktur von Kolloidteilchen mittels Röntgenstrahlen. *Nachrichten Von Ges. Wiss. Zu Gött. Math.-Phys. Kl.* 98–100 (1918).
2. Patterson, A. L. The Scherrer Formula for X-Ray Particle Size Determination. *Phys. Rev.* **56**, 978–982 (1939).
3. Coelho, A. A. TOPAS and TOPAS-Academic: an optimization program integrating computer algebra and crystallographic objects written in C++. *J. Appl. Crystallogr.* **51**, 210–218 (2018).
4. Dippel, A.-C. *et al.* Beamline P02.1 at PETRA III for high-resolution and high-energy powder diffraction. *J. Synchrotron Radiat.* **22**, 675–687 (2015).
5. Juhás, P., Davis, T., Farrow, C. L. & Billinge, S. J. L. PDFgetX3: a rapid and highly automatable program for processing powder diffraction data into total scattering pair distribution functions. *J. Appl. Crystallogr.* **46**, 560–566 (2013).
6. Cory, D. G. & Ritchey, W. M. Suppression of signals from the probe in bloch decay spectra. *J. Magn. Reson. 1969* **80**, 128–132 (1988).
7. Bruno, I. J. *et al.* New software for searching the Cambridge Structural Database and visualizing crystal structures. *Acta Cryst. B* **58**, 389–397 (2002).
8. Coelho, A. A., Chater, P. A. & Kern, A. Fast synthesis and refinement of the atomic pair distribution function. *J. Appl. Crystallogr.* **48**, 869–875 (2015).
9. Habermehl, S., Schlesinger, C. & Prill, D. Comparison and evaluation of pair distribution functions, using a similarity measure based on cross-correlation functions. *J. Appl. Crystallogr.* **54**, 612–623 (2021).
10. Habermehl, S., Schlesinger, C. & Schmidt, M. U. Structure determination from unindexed powder data from scratch by a global optimization approach using pattern comparison based on cross-correlation functions. *Acta Cryst. B* **78**, 195–213 (2022).
11. Giannozzi, P. *et al.* QUANTUM ESPRESSO: a modular and open-source software project for quantum simulations of materials. *J. Phys. Condens. Matter* **21**, 395502 (2009).
12. Lee, K., Murray, É. D., Kong, L., Lundqvist, B. I. & Langreth, D. C. Higher-accuracy van der Waals density functional. *Phys. Rev. B* **82**, 081101 (2010).
13. Hamada, I. van der Waals density functional made accurate. *Phys. Rev. B* **89**, 121103 (2014).
14. Prandini, G., Marrazzo, A., Castelli, I. E., Mounet, N. & Marzari, N. Precision and efficiency in solid-state pseudopotential calculations. *Npj Comput. Mater.* **4**, 1–13 (2018).
15. Charpentier, T. The PAW/GIPAW approach for computing NMR parameters: a new dimension added to NMR study of solids. *Solid State Nucl. Magn. Reson.* ***40**, 1 – 20 (2011).*
16. Corso, A. Pseudopotentials periodic table: From H to Pu. *Comput. Mater. Sci.* **95**, 337–350 (2014).
17. Franco, F., Baricco, M., Chierotti, M. R., Gobetto, R. & Nervi, C. Coupling Solid-State NMR with GIPAW ab Initio Calculations in Metal Hydrides and Borohydrides. *J. Phys. Chem. C* **117**, 9991–9998 (2013).
18. Harris, R. K., Hodgkinson, P., Pickard, C. J., Yates, J. R. & Zorin, V. Chemical shift computations on a crystallographic basis: some reflections and comments. *Magn. Reson. Chem. MRC* **45 S1**, S174-186 (2007).
19. Lodewyk, M. W., Siebert, M. R. & Tantillo, D. J. Computational Prediction of 1H and 13C Chemical Shifts: A Useful Tool for Natural Product, Mechanistic, and Synthetic Organic Chemistry. *Chem. Rev.* **112**, 1839–1862 (2012).
20. Gao, P., Wang, X., Huang, Z. & Yu, H. ^{11}B NMR Chemical Shift Predictions via Density Functional Theory and Gauge-Including Atomic Orbital Approach: Applications to Structural Elucidations of Boron-Containing Molecules. *ACS Omega* **4**, 12385–12392 (2019).
21. Macrae, C. F. *et al.* Mercury 4.0: from visualization to analysis, design and prediction. *J. Appl. Crystallogr.* **53**, 226–235 (2020).
